# Responsible Artificial Intelligence (AI) for Value Formation and Market Performance in Healthcare: the Mediating Role of Patient’s Cognitive Engagement

**DOI:** 10.1007/s10796-021-10136-6

**Published:** 2021-04-29

**Authors:** Pradeep Kumar, Yogesh K. Dwivedi, Ambuj Anand

**Affiliations:** 1 Indian Institute of Management Ranchi, Ranchi, India; 2 Emerging Markets Research Centre (EMaRC), School of Management, Swansea University, Bay Campus, Wales SA1 8EN Swansea, UK

**Keywords:** Artificial intelligence, Cognitive engagement, Healthcare, Market performance, Responsible AI, Value formation

## Abstract

The Healthcare sector has been at the forefront of the adoption of artificial intelligence (AI) technologies. Owing to the nature of the services and the vulnerability of a large section of end-users, the topic of responsible AI has become the subject of widespread study and discussion. We conduct a mixed-method study to identify the constituents of responsible AI in the healthcare sector and investigate its role in value formation and market performance. The study context is India, where AI technologies are in the developing phase. The results from 12 in-depth interviews enrich the more nuanced understanding of how different facets of responsible AI guide healthcare firms in evidence-based medicine and improved patient centered care. PLS-SEM analysis of 290 survey responses validates the theoretical framework and establishes responsible AI as a third-order factor. The 174 dyadic data findings also confirm the mediation mechanism of the patient’s cognitive engagement with responsible AI-solutions and perceived value, which leads to market performance.

## Introduction

In recent years, there has been an enhanced focus on artificial intelligence (AI) in various domains, to resolve complex issues (Chen, 2018; Duan et al., 2019; Dwivedi et al., 2020; 2021; Reddy, 2018). In healthcare, various forms of AI technologies have enabled the service providers to automate the process and personalize the service delivery (Ahmed et al., 2018; Brisimi et al., 2018; Shareef et al., 2021). AI has enabled the delivery of precision medicine and holds the promise of liberating patient data (Wang et al., 2018). However, these rapid advancements of AI technologies create numerous challenges and raise legitimate concerns (Sivarajah et al., 2017; Vayena et al., 2018). In effect, a responsible approach to AI has received significant attention from scholars and practitioners, to ensure fair use and sustainable impact of AI technologies (Bag et al., 2021; Balakrishnan & Dwivedi, 2021; Dubey et al., 2020; Gursoy et al., 2019; Ismagilova et al., 2020; Nishant et al., 2020; Pillai et al., 2020; 2021; Wang et al., 2020; Wearn et al., 2019). Responsible AI is characterized by ethical and accountable solutions in organizational strategies and design (He et al., 2019).

Utilization of recent AI technologies in healthcare, coupled with patient’s growing adoption of AI-enabled devices, allows collection and storage of and access to large scale data, both by healthcare providers and technology vendors (Fox & James, 2020; Mikalef & Gupta, 2021; Shareef et al., 2021). Despite the many potential benefits of such rapid technological advances in AI, its dark side calls for a responsive design and implementation as risk mitigation measures (Ahmed et al., 2018; Khalifa et al., 2019). Some common risks include security of healthcare databases and applications, violation of the end user’s privacy, and the social risks associated with uneven distribution of benefits (Fox & James, 2020; Wang et al., 2018). Especially in countries with a significant marginal population, AI increases the vulnerability of a large section of end-users. The recent report on National Strategy for Artificial Intelligence in India indicates that advancements in AI-based technologies present potential solutions to various challenges in healthcare delivery to the community (AHHM, 2017). Responsible AI is an attempt to mitigate the aforesaid risks, while simultaneously adapting to the needs of the diverse and marginalized sections of society (Ghallab, 2019; Obermeyer et al., 2019; Winter & Davidson, 2019). Although there is growing concern regarding the benefits and implementation of AI technologies, what constitutes responsible AI is still unclear.

Many studies provide evidence of the use of AI to influence clinical practices and the overall patient journey (Daugherty et al., 2019). Digital healthcare in India has improved the efficiency in processes and enhanced patient care. New health technologies such as wearable devices, growth of telemedicine, virtual reality, robotics and artificial intelligence (AI) are changing the landscape of the Indian healthcare sector (Markets, 2020). A paradigm shift in the healthcare delivery system is observed through the application of AI to radio-diagnosis, drug discovery, patient risk identification, and electronic health monitoring (Doumbouya et al., 2014; Rahman et al., 2016; Saha & Ray, 2019). Substantial growth in AI technologies has supported the healthcare service providers in basic guidance to the patients, problem-solving, and supports for various medical issues (Daugherty et al., 2019). Healthcare practitioners emphasize that AI based technologies are helpful not only in identifying the intensity of treatment, but also in classifying them into high risk or very high risk groups (Tyagi, 2019). Researchers further envisage that market, liability laws, external regulations, and internal motivation may force the healthcare industry to adopt responsible AI sooner than later (Wang et al., 2018). The demonstrable productivity of responsible AI improves market performance in healthcare (Chace, 2015; He et al., 2019). Additionally, the patients’ adoption of AI-driven solutions is expected to witness an exponential increase in the next few years (Manyika et al., 2013).

The National e-Health Authority provides evidence of the potential market for AI driven applications in India (NeHA, 2016). Several companies like Google, Microsoft and IBM are creating an AI-focused network and partnership with Indian hospitals and governments (NITI Aayog, 2016). Healthcare companies are utilizing recent technologies and AI-based solutions to capture patient’s interest and for steering new business strategies (Basu et al., 2021). According to the Future Health Index (FHI), the applications of AI in the healthcare sector in India would be worth US$ 6 billion by 2021 (FHI, 2020). As such, India is leading in the adoption of digital healthcare services and is expected to drive the healthcare market at a CAGR of 20 % by the end of 2022 (AHHM, 2017). Recent reports predict that by 2025, there would be a $520 billion opportunity from value creation through socially responsible AI, in the Indian healthcare market (Burkhardt et al., 2019; Chatterjee, 2020). Digital health technology is a pivotal pillar in delivering value-based care across the healthcare continuum in India. These levers of value creation have immense potential to increase the health expenditure aligned with market growth. Thus, the capabilities of responsible AI will drive the healthcare market and enable healthcare customers (patients) experience a dynamically different service environment through evidence-based approaches.

Pre-eminent studies in this field argue that developing responsible AI is aimed at minimizing the patent’s distrust and improving their cognitive engagement with AI-enabled technologies (Ismagilova et al., 2020; Porra et al., 2020). Recent studies have extensively examined how customer’s perspectives and the factors underlying the adoption of various newer technologies, are essential for framing marketing strategies (Khanna et al., 2012; Rana & Dwivedi, 2016). However, although the concept of cognitive engagement with socially responsible AI technologies promises to deliver significant value, many gaps still exist regarding the linkages of ‘cognitive engagement’ in the formation of a patient’s perceived value. Therefore, understanding the patient’s perception of responsible AI and the value creation process remains a vital facet of marketing. This research aims to improve such understanding of responsible AI, while considering the psychological perspective of patients and their linkages with value formation and market performance.

This study is guided by the following research questions:

1. What are the components of responsible AI in healthcare?
2. What is the impact of responsible AI on patients’ perceived value and market-performance?
3. Does cognitive engagement mediate the relationship between responsible AI and perceived value?

We conducted a mixed-method sequential approach to examine the components of responsible AI. The sample was collected from Indian healthcare systems. The first stage of the study was exploratory interviews (N = 12) to explain the relevance of the constructs under study and identify additional measurement parameters. In the second stage, a quantitative survey (N = 290) was conducted among healthcare professionals, which established responsible AI as a third-order factor. Finally, we collected dyadic data (N = 174) utilizing a survey of healthcare professionals and patients. We used PLS-SEM to test the proposed relationships. The findings highlight the complex factors of responsible AI. This study is a first step to establish the constituents of responsible AI as a third-order factor. The results of this study enrich the more nuanced understanding of how responsible AI influences the patient’s instrumental and terminal values, which in turn affect market performance. The findings of the study bridge the gap between theory and practice by clarifying how ethical concerns, technical skills, and risk mitigation factors should be implemented to design responsible AI systems. Our study confirms the mediating mechanism of cognitive engagement with responsible AI and instrumental and terminal values. The implications for researchers and practitioners are discussed, emphasizing that healthcare service providers need to design responsible AI to develop value propositions and improve market performance.

## Literature Review and Hypothesis Development

In recent years, AI has achieved an accelerated momentum to deliver the best possible outcomes and is increasingly prevalent in business and society (Sharma et al., 2016). Proponents of AI point out its affording tremendous potential to affect every sphere of human life and activity. Although artificial intelligence technology is inherently complex, integrated, and spread across multiple organizations, the capabilities of AI to drive revenues and profitability have opened a wealth of opportunities across the field (Bichinadaritz & Marling, 2006; He et al., 2019; Shukla & Sushil, 2020).

### AI for Healthcare

In healthcare, AI has a crucial role in improving the clinical outcomes and overall efficiency of managerial activities. Prior studies have outlined how AI can improve healthcare delivery by augmenting human abilities, supporting mental health, and precise diagnosis (WHO, 2020b; Wu et al., 2021). The AI technology allows us to gain information, process it, and generate a well-defined output for medical professionals (Reddy, 2018; Wang et al., 2018). AI technologies possess data-mining and pattern recognition capabilities that enable the prediction, diagnosis and treatment (Kok et al., 2013). Early attempts to apply AI technologies in medicine were intended to replicate the functions of the human brain and set up a rule-based system to assist medical reasoning (He et al., 2019; Warwick, 2013). Further developments in AI were focused on replicating the intellectual function of the physician. However, the modern products of AI technologies are overcoming the various limitations of clinicians and complexities in the care process (Reddy, 2018; Wang et al., 2020). The application of AI converts analytical insights into cognitive engagement solutions that enhance diagnosis, improve predictive interventions, and optimize clinical productivity (Fox & James, 2020; Porra et al., 2020; Wang et al., 2018).

AI technology is capable of accelerating the shift from traditional hospital settings to customer-focused care sites like ambulatory surgical centers, retail clinics, and home care (Barello et al., 2016; Manyika et al., 2013). Further, home care and wearable devices may lower the overall health costs by 20 to 32 % (Saha & Ray, 2019; Sultan, 2015). AI-enabled technologies have opened up a new vista of home infusion and observation care models, which are expected to grow by more than 18 % over the next five years (OECD, 2019). Specifically, it is becoming easier to understand patient’s health patterns, with improvements in predictive analytics, enabling clinicians to remotely monitor patients under home care or through connected devices. Thus, healthcare professionals have benefitted from improved prevention, diagnosis, and care processes.

On the other hand, the inherent power of AI creates substantial threats to organizations, stakeholders, and the industry supply chain (Obermeyer et al., 2019; Sivarajah et al., 2017). Past studies have reported that the more advanced the AI, the greater are the challenges and threats it poses humanity. Risks associated with AI include (but are not limited to) the safety of critical AI applications, security and privacy of user data, and social risks (Joubert et al., 2021; Porra et al., 2020; Sharma & Sharma, 2019; Vellido, 2019). For instance, the collection of patient data by AI algorithms raises serious issues of privacy invasion, transparency, and information leakage (Fox & James, 2020). Wang et al. (2020) catalogue several risks for healthcare deliveries like compromising transparency standards, neglecting fair clinical deployment, and ignoring the algorithmic biases. Thus, the utilization of such advanced technologies has exposed individuals to many risks at various levels of data collection and processing. Consequently, there has been an increasing concern about the ethical issues and legitimacy associated with AI technologies (Deven & Joshua, 2017; Lui & Lamba, 2018). In recent years, tremendous concern about AI has been a expressed in various forums, corporations, and government departments (NAH, 2020; NeHA, 2016; Thomas, 2020) in India. The Medical Council of India has implemented data protection regimes to regulate ‘private data’ (MCI, 2016). Consequently, a shift in focus has been observed to the development and implementation of AI technologies that are socially responsible.

### Responsible AI in Healthcare

Wang et al. (2020) define responsible AI as “the integration of ethical and responsible use of AI into the strategic implementation and planning process.“ Responsible AI primarily aims to design ethical, transparent, and accountable solutions (Fox & James, 2020; Shaikhina & Khovanova, 2017; Winter & Davidson, 2019). To Abosaq (2019), responsible AI is a tool helpful for organizations to improve trust and minimize privacy invasion. Past studies highlight the importance of investigating ethical considerations, technical skills concerning data and algorithms, and risk mitigation strategies, in leveraging AI developments (Shaikhina & Khovanova, 2017; Zink & Rose, 2020; Zuboff, 2015). Several studies have furthered this discussion of responsible AI and suggest that different technical and analytical skills are required to build and use AI responsibly (Chatterjee, 2020; Gupta & George, 2016; Hung et al., 2007). Technical skills should focus on the alignment of stakeholders’ expectations for the use of data. The magnitude of risk often dictates the importance of risk mitigation strategies. The term responsible AI encompasses all these risk-mitigating activities. Such strategies will need wider collaborations (Bengatson & Kock, 2000; Campbell, 2007). Burkhardt et al. (2019) argue that it is generally the external regulations and internal motivation that force a typical AI firm to strive for safer AI products. Such efforts, however, do not always lead to increased profit or reduced litigation cost. Hence, it is crucial to identify factors that can facilitate co-operative development of responsible AI, rather than a competitive battle in the market. Trust among stakeholders and high shared gains from cooperation are a couple of such factors (Hung et al., 2007). Several researchers opined that AI-based systems are intrinsically autonomous, interactive, and adaptable (Chace, 2015; Hsu et al., 2021; Shaikhina & Khovanova, 2017). While exploring AI, Wang et al. (2020) integrate it with the ART principles: accountability, responsibility and transparency. Accountability would ensure that all the decisions and outcomes are justified to all the stakeholders. These decisions should be derivable from the original set of data or information used. Responsibility would ensure that all the developers and researchers are aware of their commitment, more so in cases where the impact of AI on society is significant and direct. Finally, transparency would ensure that the mechanism through which AI makes a particular decision is described to the stakeholders, who should be able to further inspect and reproduce the process of decision making. This is a significant departure from the existing, more popular black-box approach of AI (Barrat, 2013; Chopra, 2019; Kok et al., 2013).

Though these concepts of accountability, responsibility, and transparency may be considered essentials of a responsible AI, their applicability dictates acceptance boundaries. For instance, Wang et al. (2018) hold explainability to be core to responsible AI. However, the expectations of a typical end-user, especially in the healthcare domain, might vary. The authors explain this with an example of a healthcare application that predicts skin cancer with the help of images. In this context, most patients would care less about the model’s nuances and more about its accuracy of prediction and recommendations. Thus, the same principles need to be applied differently in diverse application areas. Many studies further point to the importance of appropriate data acquisition, data-set suitability, fairness of AI outputs, and regulatory compliance and engagement, in the context of leading an organization to use responsible AI (Burkhardt et al., 2019; Duan et al., 2019; Dwivedi et al., 2021; Grover et al., 2020; Fuentes, 2015; Wearn et al., 2019). Al-quaness et al. (2020) utilized COVID-related responses to highlight the gaps and potential pitfalls in the existing AI approach. These include algorithm bias and discrimination, adverse data impact, lack of process transparency, and model interpretability. According to the authors, the challenges to autonomy, privacy, and public trust are also evident in various COVID-related contact tracing applications. In this context, it was also observed that some of these solutions tend to shift focus from supporting effective medical response to the concerns of mass surveillance and politics of public distrust. A responsible AI approach in such a context would emphasize reproducibility, replicability, and transparency. Numerous studies further propose key steps for responsible AI, including advice to open science, share data responsibly, adopt ethical principles, generate public trust, foster equitable innovation and protect the interests of the vulnerable (Dwivedi et al., 2021; GDPR, 2019; Modjarrad et al., 2016).

**Fig. 1:**
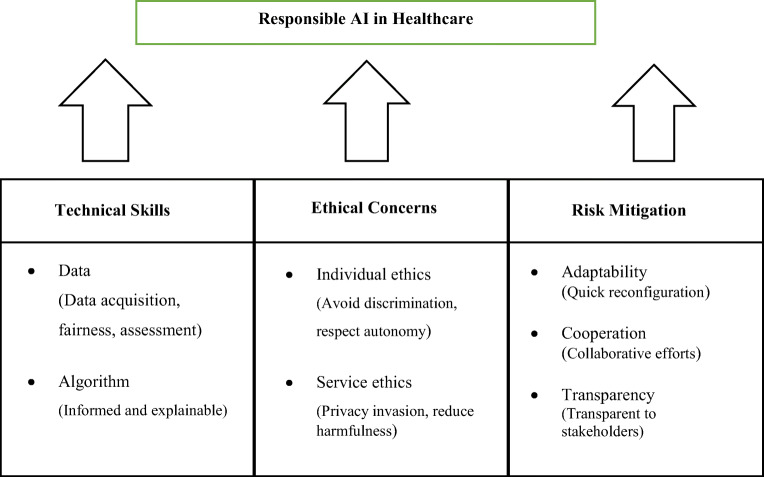
Responsible AI in healthcare: Author’s preliminary conceptualization

It is evident from the above discussion that responsible AI is a multi-dimensional construct focusing on technical challenges and skills, ethical concerns, and risk mitigation. The critical appraisal of the existing literature thus reveals the constituent elements of responsible AI. This study conceptualizes responsible AI as a third-order factor (see Fig. 1).

In healthcare, value creation is recognized as an essential element of the service provider’s competitive strategy (Da Silva et al., 2015; Sergio, 2015). Management researchers have identified that actively managing value and devising ways to develop value propositions are crucial for sparking growth (Chen et al., 2017).The means-end theory (Rokeach, 1973) states that personal values include instrumental values and terminal values, holding that personal values influence individual behavior. According to Parry (2001), the means-end theory suggests that personal values are the end that people seek, while the means help achieving such end. In the current study context, the means are patients’ experience of positive consequences of responsible AI. Based on past studies (Amit & Zott, 2001; De Sarbo et al., 2001), we posit that patients perceive different benefits from these technologies that affect instrumental and terminal values. Responsible AI makes patient interactions more convenient and trustworthy. These platforms deliver the financial benefit of cost reduction and psychological benefits of variety and creating instrumental value of cheerfulness. Besides, these platforms provide functional benefits through reduced hospitalization and a hassle-free care process. Thus, the patients perceive the instrumental values of cheerfulness, self-control, and responsibility. Chen et al. (2017) found that experiential benefits create customer terminal value. Easy access and risk reduction by engaging with responsible AI infuses confidence in patients in sharing personal information. Responsible AI solutions deliver psychological benefits of risk reduction, making patients feel happy and comfortable. Risk reduction by utilizing AI technologies creates the terminal value of nostalgia (Almquist et al., 2016). Magids et al. (2015) explored emotional-connection-driven opportunities and found that these technologies make sense of freedom and belongingness, thus driving customer behavior and creating terminal value. Thus, responsible AI provides benefits that are perceived as offering more terminal value. Therefore, it is hypothesized that:

- H1: Responsible AI in healthcare has a positive influence on perceived instrumental value.
- H2: Responsible AI in healthcare has a positive influence on perceived terminal value.

### Value Formation and Market Performance

Market performance is defined as the capability to enter new markets and introduce new services faster than the competitors (Ravichandran & Lertwongsatien, 2005). Wang et al. (2012) posit that market performance indicates a more significant market share and success rate than the competitors. Several researchers and practitioners argue that customer value is an essential element of a firm’s market performance. According to Chen et al. (2017), customer value management is a strategic tool to enhance market performance. Many studies indicate a significant relationship between perceived value and market-based outcomes (Javalgi et al., 2005; Johnson et al., 2003; Ravichandran & Lertwongsatien, 2005). Lusch and Nambisan (2015.p.159) stress that “the organization’s performance will fall or rise based on the perceived value.“ Many studies have shown that the customer’s perceived value is a critical measure of the firm’s market performance (Brozovic et al., 2016; Gronroos & Ravald, 2010). According to Chen et al. (2017), a customer’s perceived value is a subjective indicator of a firm’s market performance. Ravichandran and Lertwongsatien (2005) showed that customer value improves market share and enables firms to remain competitive in the market. Researchers believe that the several value characteristics (e.g., courage, cheerfulness, responsibility, and self-control) perceived by the customers indicate the firm’s success and thus affect market performance. Similarly, many researchers posit that customer’s perceived terminal values (e.g., pleasure, comfort and self-respect) help in the introduction of new services and affect market performance. Existing studies aver that perceived value remains a vital issue for healthcare services (Da Silva et al., 2015; Nair et al., 2013). The overall market performance of a healthcare firm is a function of perceived values. Patients’ evaluation of a specific service attribute is likely to affect their relationships with the service provider, which affects the firm’s market performance. Past studies (Bate & Robert, 2007) indicate that the beliefs and impressions customers hold toward a service provider form a brand image, which influences the latter’s market performance. Likewise, the perceived instrumental and terminal values of socially responsible AI technologies being utilized in various care procedures generate the best performance outcomes.

Thus, we postulate that:

- H3: Patients’ perceived instrumental value positively influences market performance.
- H4: Patients’ perceived terminal value positively influences market performance.

### Cognitive Engagement with Responsible AI

Cognitive engagement is connected to what the patient knows, understands, and how he/she makes sense of the disease, its treatments, its possible development, and its monitoring (Serino et al., 2014). As a significant part of the overall learning and experience process, cognitive engagement enables patients to immerse in in-depth learning processes situated in realistic healthcare problems (Gen, 2015). AI technologies in healthcare aim to increase patients’ cognitive engagement with those applications and tools providing personalized care (Reddy, 2018). A recent study (Singhal & Carlton, 2019) states that AI technologies are designed to help patients’ to improve their health conditions, make informed decisions, and engage effectively and efficiently with the healthcare system. Graffigana et al. (2015) described the dynamic nature of cognitive engagements as a psychosocial phenomenon resulting from the conjoint beliefs, goals, and epidemiology enactment of individuals towards their health condition and management. Extant literature also indicates that the particular benefits and value patients perceive concerning any given technology for their care process, influence their cognitive engagement (Agarwal et al., 2013; Bashshur et al., 2011; Coulter & Ellins, 2007; Hibbard et al., 2007).

The healthcare sector describes cognitive engagement mainly as a critical factor in obtaining adequate and customized disease management plans for patients (Linn et al., 2011). The importance of involving patients in learning and utilizing new technologies lies in fostering their self-management skills (Faber et al., 2017). Therefore, cognitive engagement in the context of responsible AI in healthcare is explained as a pivotal element in legitimizing the patient’s expression of physical and emotional needs, thus better orienting professional interventions (Marano & Nicolantonio, 2015). Responsible AI technologies offer valuable devices and platforms to facilitate patient activation and engagement in self-care and preventive behaviors (Gambhir et al., 2016). The perceptions of reasonable AI encourage patients to engage with them actively. For instance, promises to prevent the harmful effects, fairness in dealing with patients and their data, and transparency in the processes will increase patients’ cognitive engagement with such technologies. Thus, cognitive engagement with responsible AI is oriented by a broader vision of healthcare that goes beyond the organizational boundaries of healthcare settings. Responsible AI technologies seek to learn more about health concerns and collaborate with physicians in making treatment decisions, and communicate and share information with them (Kim, 2015; Wimmer et al., 2016).

**Fig. 2:**
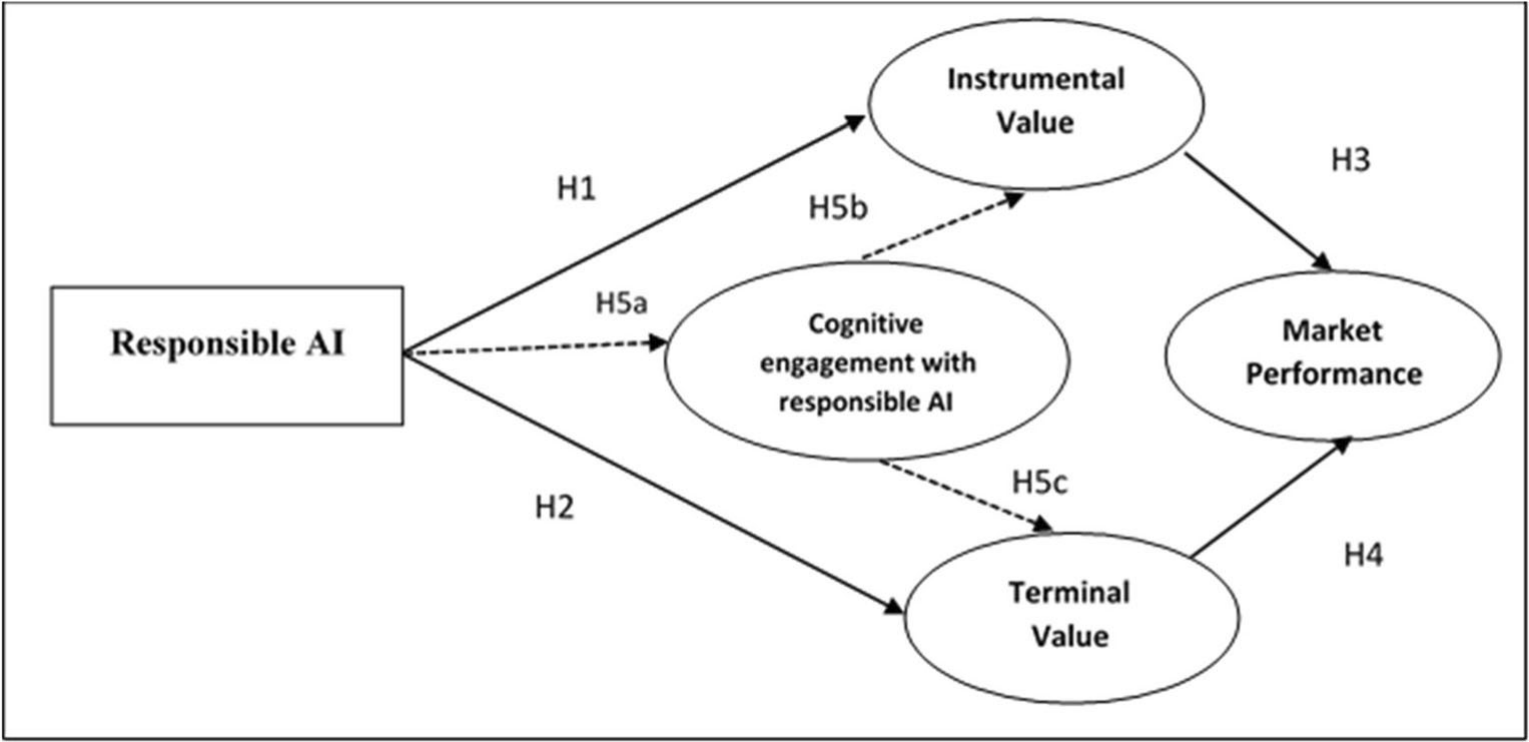
Proposed conceptual model.(Source: author’s conceptualization)

Cognitive engagement with responsible AI technology has several positive consequences, one of which is perceived value (Chen et al., 2017). Numerous researchers have established significant relationships between cognitive engagement and perceived value. For example, Brodie et al. (2011) argued that cognitive engagement with particular circumstances and service environment leads to higher perceived values. Some studies have found that cognitive engagement is linked to the assurance of instrumental and experiential values and pleasure and satisfaction (Amit & Zott, 2001). Researchers have also commented on the propensity of cognitive engagement to strengthen perceived value (Eggers & Kaplan, 2013). In addition, the customer’s evaluation of the consumption experience is fundamental in creating instrumental and terminal values (Zeithmal, 1988). In healthcare, patients perceive several benefits by the consumption of various service products that are adjacent to their social and self-perception, which in turn affects their ‘value-in-use’ (Gronroos & Gummerus, 2014). It has also been established that firms essentially need to focus on customer engagement, to add an array of values to the service products. Recent studies offer cognitive engagement with service products as a unit of analysis for value creation (Chen et al., 2017). Patient benefits derived from cognitive engagement with responsible AI technologies and artifacts are essential for perceived values. Cognitive engagement with responsible AI technologies forms the preferences for the process of care and channelizes the instrumental and terminal values by the rendered services. In this context, it appears that cognitive engagement by patients with responsible AI technologies corresponds to their perceived instrumental and terminal value (see Fig. 2).

Hence, we postulate that:

- H5: Cognitive engagement with responsible AI mediates the relationships between Responsible AI – Instrumental Value and Responsible AI – Terminal Value.

## Methodology

**Fig. 3:**
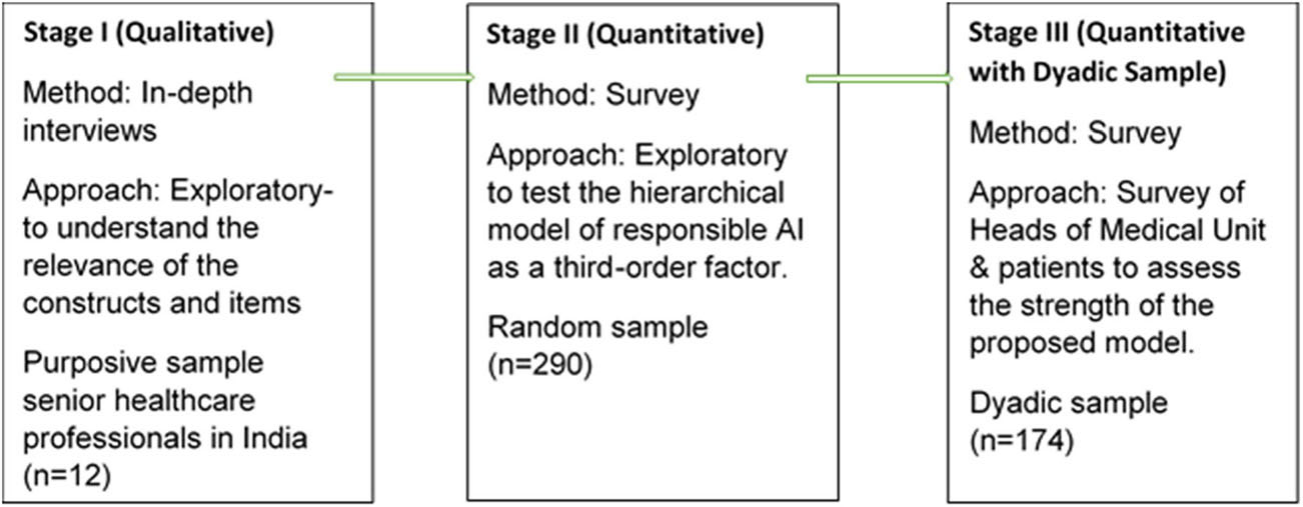
Research design

To develop more robust inferences, extant literature in the IS discipline calls for a mixed-method study (Venkatesh et al., 2013), which adopts a sequential mixed method approach and collects samples from both ends. Given the complex nature of healthcare deliveries, this study argues that a mixed-method approach offers the potential to enhance understanding of the phenomena under study. Thus, the study’s objective of illustrating the dynamics of responsible AI is aligned with the application of mixed methods. As indicated in Fig. 3, this study employs a three-stage sequential design (Fox & James, 2020), with data from each stage informing the next stage (Creswell, 2006).

### Qualitative Study

In the first stage (Study 1), an exploratory qualitative study was conducted with in-depth interviews. A purposive sampling strategy was utilized to recruit the participants. The sampling criteria ensured the likelihood of healthcare professionals (senior medical officers, senior nursing staff, and technical officers) expressing their understanding of the constituents of responsible AI in healthcare. The criteria for the interviewees were experience in and knowledge of responsible AI. The exploratory interviews emphasized the participants’ perception of responsible AI and their views as to the constituents and consequences of responsible AI. The interview guide was prepared from the concepts derived from the extant literature. We utilized a few open-ended questions (Appendix Table 9) to explore the concepts under study and identify the additional items to establish responsible AI constructs. A total of 26 interviews were conducted in multiple rounds (Appendix Table 10). Typically, interviews were conducted in the respective offices of the respondents and lasted between 40 and 60 min.

We analyzed the transcriptions utilizing framework analysis (Harrison & Reilly, 2011). The exploratory interviews confirmed the relevance of the concepts and constructs drawn from the literature. Respondents expressed their perceptions of various facets of responsible AI (Table 1). Two of them described the technical issues involved with responsible AI, explaining how the data and algorithm are sensitive issues for responsible AI. Research has shown that technical skills related to data set acquisitions and processing are crucial in the design of AI systems (Leslie, 2019). Evidence suggests that choosing the right data and the right algorithms delivers a fair outcome (Amoore & Raley, 2017; Deven & Joshua, 2017).

Further, the algorithms should be robust and better informed. Respondents substantiate the data- related factors (Gupta & George, 2016) and emphasize the use of relevant and quality data. Past studies have emphasized the importance of aligning with the stakeholder’s expectations for the use of their data (Hota et al., 2015). Two respondents expressed the patients’ concern of their data being manipulated and of privacy invasion issues as service ethics, remarking that they were trying to minimize privacy invasion to build a level of trust. In the exploratory interviews, respondents revealed potential threats of ethical issues and their impact on a responsible AI system. For instance, they expressed concern for the safety of human lives in robotic surgery. Shaikhina and Khovanova (2017) posited that AI could obtain personal information that may give rise to ethical issues. The interview analysis confirms various risk management strategies as a vital factor of responsible AI. Three respondents agreed that adaptive capacity and transparency are essential to design responsible AI systems and artifacts and that responsible AI developments can be visualized as a collective action problem with risk mitigation, requiring successful coordination with different activities.

**Table 1:** Exploratory interviews

Sl. No.	Constructs under Study	Interview responses
1	Data	“Huge amount of patient data is generated at various levels. Healthcare systems have started maintaining data infrastructure both through vendors and in-premise. Utmost important is to be sensitive about patient data….The continuous analysis in a systematic way as we express our responsiveness toward the patient and community in general. We advise the vendors to maintain relevant data. In fact, we have check-ins to ensure fairness. The skills for data handling should ensure a great focus on accuracy”. *Medical Director, 61, Male*
2	Algorithm	“For example… If a particular application is used for predictions of skin disease or cancer, the patients are only concerned about the recommendation of the model or about the prognosis. The technical skills of the developers must ensure the prevention of malfunctions. The expertise must not only be in performance …the computer programs and algorithms…. rather they should be sensitive to human lives and values”. *Medical IT Officer, 49, Male*
3	Individual Ethics	“The most significant factor in the current AI based systems is how a professional regards to moral values. The security and privacy of patient data are highly dependent upon the medical professional’s individual ethics. As we join this profession, we take oaths… I will keep it secret, I will consider all things to be private. I would say…While utilizing AI…This oath should be kept in mind”. *Medical Dean, 58, Female*
4	Service Ethics	“The medical service has its own considerations regarding the patient-related data or its harmful effect. We have an ethics committee to ensure the fair execution of services and medical records. We should attempt that AI can be an ethical producer and satisfy the patient”. *Chief Operating Officer, 52, Male*
5	Adaptability	“The AI solutions must not be a rigid system. It must consider the changes from time to time as per the emergent needs. We often discuss with the medical IT department and our technical service providers regarding the problems or the other effects. The solution must incorporate the changes and should be quickly reconfigured, particularly when some harmful effects are reported.“ *Medical Director, 61, Male*
6	Cooperation	“Such advanced technology is a group effort. Of course, we are at a nascent stage. We don’t claim to be technology experts; however, many of us are now skilled. Our technology vendors, the government departments, the medical council, the other technical societies- all work together so that the various risks of AI can be fully understood, protected by laws, and collaborative efforts can be made to reduce the risks”. *Medical Dean, 58, Female.*
7	Transparency	“While utilizing AI technologies in medical care, it must explain the mechanisms and assumptions… all the implementations should be done in a way that is known to the patients or users. All involved parties should be taken obligations to provide safety to all the human being who is associated with the AI, directly or indirectly- in a way, and the understandable explanations should be there”. *Medical IT Officer, 49, Male*

The interviews revealed four items of the various constructs utilized in the next stage measurement scale, the quantitative study. Consequently, we added four measurement items- (DD2, Alg1, SRE1, Cop2) in our model to conduct the survey (Appendix Table 11).

### Stage II: Quantitative Assessment

A measurement scale was constructed for the quantitative assessment of the proposed model. The measurement parameters were adopted from the extant literature, and four items were the outcome of exploratory interviews (Appendix Table 11). The scale items were adapted from previous studies and modified to suit the context of the study and ensure face validity. A pre-test of the survey instrument was administered to seven academicians and six healthcare professionals, to establish the questionnaire’s content validity. We also followed the recommendations of Malhotra et al. (2006) while designing the questionnaire, to reduce the effects of common method bias (CMB). We provided brief descriptions of the scale items and also ensured the anonymity of the respondents. The final scale (five-point Likert-scale with 1 = strongly disagree to 5 = strongly agree) for the survey (Study I) consisted of seven constructs and 26 items.

We conducted a survey of healthcare professionals in India, consisting of all the first-order constructs of responsible AI (seven constructs). The sampling frame was the top five performing hospitals in India. The inclusion criteria were bed strength, the volume of patients, and healthcare firms’ market performance. We approached the potential respondents (Table 2) through email and requested them to participate in the survey. A total of 290 valid responses were collected (55.17 % male, with more than 77 % between 28 and 48 years). The survey participants first responded to items about their experience with AI-enabled tools and platforms. A further explanation on scales was provided to ensure clarity and accuracy.

**Table 2:** Sample characteristics

Demographic variables	Frequency	(%)
Gender		
Male	160	55.17
Female	130	44.82
Current role		
Doctor	110	37.93
Nurse	85	29.31
Para-medical staff	16	19.31
Medical IT staff	52	04.13
Others	27	09.31
Education		
Post Graduate	150	51.72
Graduate	60	20.68
Others	80	27.58
Experience		
< 5 yrs.	40	13.79
5 To 10 yrs.	65	22.41
10 To 15 yrs.	78	26.89
15 To 20 yrs.	22	07.58
> 20 yrs.	85	29.31

#### Hierarchical Model Specification

**Fig. 4:**
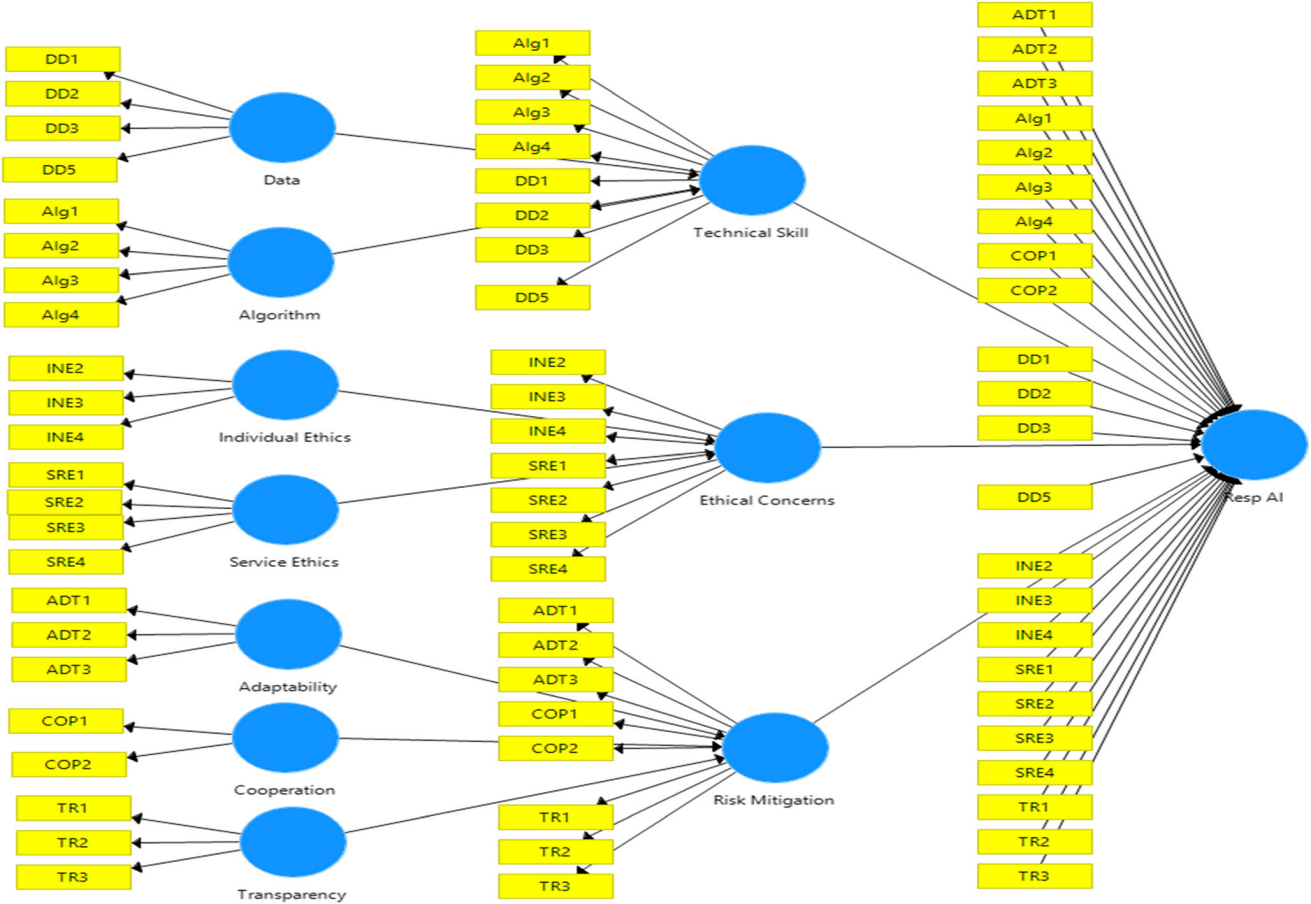
Hierarchical model specification - responsible AI

We first specified the hierarchical model, which represents the relationships between the higher-order constructs, sub-dimensions, and the measurement indicators. We constructed the model indicating the first and second-order constructs as reflective (Mode A), based on the related studies and subsequent conceptualization. The third order was constructed as formative (Mode B). To depict the hierarchical model in PLS-SEM, we first constructed the first-order latent variables and connected them to their corresponding indicators (cf. Gupta & George, 2016).The repeated item indicator approach recommended by Hair et al. (2016) was utilized to form the second-order latent constructs. The third-order factor (RespAI) was constructed by repeating the indicators of all the first-order factors (Fig. 4).

The partial least squares (PLS) approach to SEM (PLS-SEM) is used for the estimation of a complex and hierarchical model with the assumptions of soft modeling (Vinzi et al., 2010). We applied PLS path modeling to avoid the limitations of sample size and distributional properties (Sharma, 2019). Smart PLS3 software was used to conduct a non-parametric bootstrapping procedure with 5,000 re-samples and estimate the model (Ringle et al., 2017). We followed higher-order modeling procedures to establish the third-order construct (Resp AI) and developed its valid measurement instrument (Hair et al., 2016; Tenehaus et al., 2005).

#### Results

The measurement or outer model validation is the first step in PLS-SEM analysis (Sharma et al., 2017). The measurement model’s assessment consists of indicator reliability, internal consistency (composite reliability), convergent validity, and discriminant validity. Outer loadings of all the first-order reflective constructs were statistically significant. Cronbach’s (1951) alpha and the composite reliability values (CR) were above the recommended values of 0.7 (Table 3). The construct’s convergent validity was established, as the average variance extracted (AVE) values were above the recommended value of 0.5 (Hair et al., 2016). We dropped three items of the scale (DD4, INE1, and COP3) due to inadequate loadings. The discriminant validity of first-order constructs was also established as follows (Table 4). First, AVE’s square root was greater than its highest correlation with any other construct (Fornell & Larcker, 1981). Second, each construct’s outer loadings were greater than its cross-loadings with other constructs (Hair et al., 2016).

**Table 3:** Study II (Reliability and validity indices)

	Outer Loadings	t-value	VIF	CR	AVE
Data				.802	0.504
DD1	0.754	22.87	1.358		
DD2	0.739	22.918	1.264		
DD3	0.672	13.546	1.214		
DD5	0.672	10.912	1.242		
Algorithm				0.824	0.545
ALG1	0.765	23.782	1.42		
ALG2	0.756	26.929	1.384		
ALG3	0.695	24.388	1.282		
ALG4	0.72	26.322	1.362		
Individual ethics				0.777	0.538
INE2	0.727	21.858	1.384		
INE3	0.761	26.297	1.219		
INE4	0.712	20.891	1.155		
Organizational ethics				0.809	0.515
SRE1	0.747	25.204	1.333		
SRE2	0.723	21.227	1.302		
SRE3	0.682	15.78	1.229		
SRE4	0.717	22.192	1.137		
Adaptability				0.764	0.519
ADT1	0.707	17.766	1.161		
ADT2	0.764	28.344	1.326		
ADT3	0.687	19.736	1.096		
Cooperation				0.807	0.676
COP1	0.802	30.494	1.143		
COP2	0.842	46.349	1.244		
Transparency				.774	.533
TR1	0.702	21.111	1.529		
TR2	0.729	21.07	1.190		
TR3	0.7759	28.418	1.219

**Table 4:** Test for discriminant validity

	Adaptability	Algorithm	Cooperation	Data	Ethical Concerns	Individual Ethics	Resp AI	Risk Mitigation	Service Ethics	Technical Skill	Transparency
Adaptability	0.72										
Algorithm	0.55	0.735									
Cooperation	0.415	0.502	0.822								
Data	0.27	0.302	0.158	0.71							
Ethical Concerns	0.182	0.112	0.196	0.016	0.644						
Individual Ethics	0.179	0.084	0.177	0.002	0.844	0.733					
Resp AI	0.71	0.761	0.63	0.448	0.577	0.49	NA				
Risk Mitigation	0.834	0.659	0.738	0.267	0.232	0.203	0.844	0.617			
Service Ethics	0.149	0.11	0.172	0.026	0.923	0.572	0.529	0.208	0.718		
Technical Skill	0.538	0.883	0.447	0.713	0.075	0.063	0.78	0.616	0.069	0.581	
Transparency	0.590	0.565	0.505	0.214	0.195	0.149	0.727	0.875	0.19	0.521	0.73

*Diagonal elements are the square root of AVE.

The structural model was estimated using the bootstrapping procedure with 5000 resamples (Hair et al., 2016). The bootstrap test indicated that the loadings were highly significant. The multicollinearity for each construct’s predictors was checked using VIF values (Table 3), which were lower than 5 as recommended by Hair et al. (2016).

**Table 5:** Path Co-efficient: Third Order factor validations

Direct Impact	Path Coefficient	T Statistics	P Values
Adaptability -> Risk Mitigation	0.429	20.145	0.000
Algorithm -> Technical Skill	0.702	15.613	0.000
Cooperation -> Risk Mitigation	0.33	17.565	0.000
Data -> Technical Skill	0.527	12.454	0.000
Ethical Concerns -> Resp AI	0.455	30.28	0.000
Individual Ethics -> Ethical Concerns	0.486	20.366	0.000
Organizational ethics -> Ethical Concerns	0.634	25.199	0.000
Risk Mitigation -> Resp AI	0.442	30.685	0.000
Technical Skill -> Resp AI	0.463	31.486	0.000
Transparency -> Risk Mitigation	0.453	22.5	0.000

**Fig. 5:**
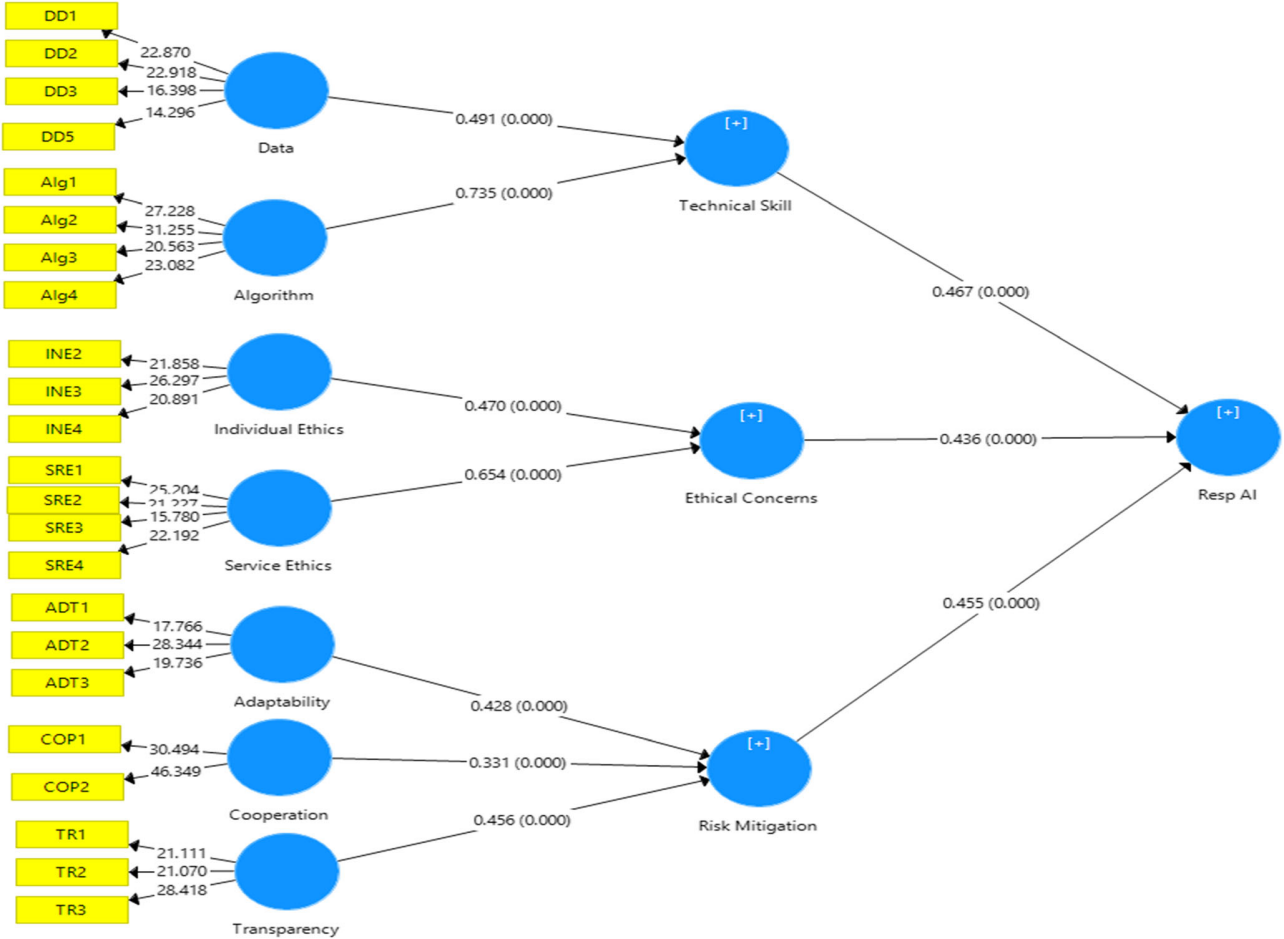
Bootstrapping – responsible AI as a third-order factor

We evaluated the hierarchical model next. The study first intended to establish the three second-order factors – Technical Skills, Ethical Concerns, and Risk mitigation. The indicator weights of the second-order constructs – (1) Data (β = 0.491, t = 12.331, p = 0.000) and Algorithm (β = 0.735, t = 17.069, p = 0.000) on ‘Technical Skill’ (2) Individual ethics (β = 0.470, t = 21.441, p = 0.000) and Service-ethics (β = 0.654, t = 28.041, p = 0.000) on ‘Ethical concerns’ and (3) Adaptability (β = 0.428, t = 21.065, p = 0.000), Co-operation (β = 0.331, t = 18.472, p = 0.000), and Transparency (β = 0.456, t = 23.63, p = 0.000), on ‘Risk mitigation’ factors- were significant. Further, the indicator weights of the third-order construct – Technical Skill (β = 0.467, t = 35.517, p = 0.000), Ethical Concerns (β = 0.436, t = 31.020, p = 0.000) and Risk mitigation (β = 0.455, t = 36.483, p = 0.000) were statistically significant (Fig. 5). We assessed the structural model by path co-efficient, t-statistics, and p-values (Table 5). Further, blindfolding procedures were utilized to obtain predictive relevance (omission distance = 7). Results demonstrated positive Q^2^ (construct cross-validated redundancy) values for Technical Skill (Q^2^ = 0.3299), Ethical Concerns (Q^2^ = 0.409), Risk Mitigation (Q^2^ = 0.375), and Resp AI (0.204), thus indicating a satisfactory predictive relevance. Finally, the overall model fit was assessed by using standardized root mean square (SRMR) residuals as an index for model validation (Henseler et al., 2014). The PLS results indicated an SRMR value of 0.058, which is less than the threshold of 0.10 (Hair et al., 2016).

### Study III: Quantitative with Dyadic Sample

After establishing the Resp AI construct as a third-order formative factor, the next stage of the study intended to assess the strength of the proposed relationships. Data for study III were collected from those in charge of various medical units and patients. A separate survey was created by adding questions of market performance construct and the refined set of items for Resp AI. We checked whether any respondent from the initial survey was also involved in this data collection stage. Further, these respondents were requested to provide the name and contact details of at least two or three patients. These participants (patients) had been using AI-enabled tools or other platforms for the last one to two years (e.g., - ‘FitBit’, wearable ECG, chat boats). Thus, we further found the respondents’ list, which consisted of the other end of the healthcare provider-patient dyad (Gooty & Francis, 2011). The patients’ questionnaire consisted of three constructs with four measurement items: cognitive engagement, instrumental value, and terminal value. Snowball sampling was utilized to reach the other end of the healthcare provider-patient dyad (Gummenson, 2005). Thus, a sample of 174 dyads was formed.

We further utilized PLS-SEM to assess the strength of the relationships depicted in the hypothesized model (Fig. 2). A similar method was followed to evaluate the outer loadings of the first order constructs, which were found to be significant (> 0.7), and construct reliability was also established (Table 6). For reflective constructs, the AVE values were greater than 0.5. The discriminant validity results are given in Table 7.

**Table 6:** Study III (Reliability and validity indices)

	Outer Loadings	t-value	VIF	CR	AVE
Cognitive engagement				0.810	0.621
Cog1	0.704	17.096	1.414		
Cog2	0.757	24.34	1.465		
Cog3	0.696	17.919	1.239		
Cog4	0.713	18.331	1.276		
Instrumental value (INV)				0.824	0.632
INV1	0.706	16.859	1.329		
INV2	0.730	18.207	1.439		
INV3	0.728	20.215	1.303		
INV4	0.773	26.121	1.471		
Terminal value (TNV)				0.815	0.589
TNV1	0.678	14.256	1.256		
TNV2	0.727	16.966	1.412		
TNV3	0.726	19.568	1.277		
TNV4	0.765	25.126	1.425		
Market Performance				0.813	0.525
MKT1	0.662	15.445	1.327		
MKT2	0.810	30.678	1.367		
MKT3	0.717	16.867	1.363		
MKT4	0.695	15.146	1.229

**Table 7:** Tests for discriminant validity

	Cog Eng	INV	MKP	Resp AI	TNV
Cog Eng	0.718				
INV	0.456	0.735			
MKP	0.423	0.404	0.723		
Resp AI	0.561	0.514	0.463	NA**	
TNV	0.401	0.883	0.39	0.446	0.725

*Diagonal elements are the square root of AVE

**Resp AI is a formative construct

**Fig. 6:**
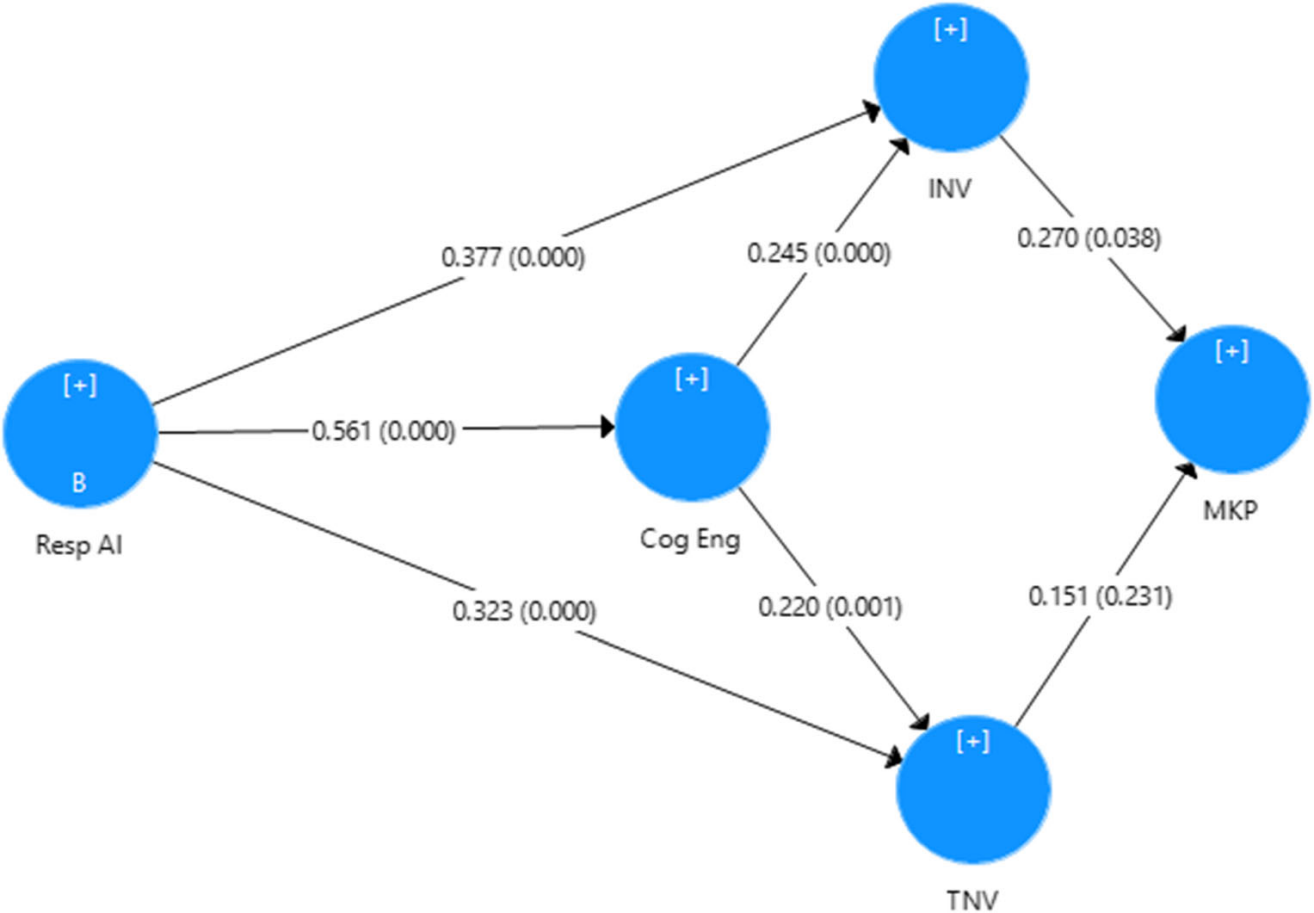
Bootstrapping – validated conceptual model. *path coefficient and p-values

The structural model was estimated through bootstrapping. The direct impact of Resp AI on INV (β = 0.531, t = 14.665, p = 0.000) and TNV (β = 0.465, t = 12.257, p = 0.000) was significant. Thus, the hypothesis H1 and H2 are supported (Table 8). Mediation analysis was performed as recommended by Baron and Kenny (1986). The strength of this relationship was suppressed (Table 3) when the mediating variable (Cog Eng) was introduced. However, the impact of Resp AI on Cog Eng (β = 0.561, t = 15.427, p = 0.000) and the relationships of Cog Eng-INV (β = 0.245, t = 3.708, p = 0.001) and Cog Eng-TNV (β = 0.220, t = 3.413, p = 0.001) were significant. Moreover, the indirect relationships Resp AI-INV (β = 0.205, t = 3.075, p = 0.002) and Resp AI-TNV (β = 0.205, t = 3.075, p = 0.002) were also significant. Thus, the mediation mechanism (H3) of Cog Eng was established. Finally, the impact of INV on MKP (β = 0.0.270, t = 2.071, p = 0.038) was significant, while that of TNV on MKP (β = 0.151, t = 1.198, p = 0.231) was insignificant (Fig. 6). Thus, hypothesis H4 was supported, while H5 was not supported.

**Table 8:** Hypothesis testing

Direct impact	Standardized direct effect	Standard error	t value	p-value	Hypothesis testing
Resp AI -> INV (H1) *	0.377	0.064	5.866	0.000	*Supported*
Resp AI -> TNV (H2)*	0.323	0.061	5.280	0.000	*Supported*
INV -> MKP (H3)	0.151	0.126	1.198	0.231	*Supported*
TNV -> MKP (H4)	0.27	0.131	2.071	0.038	*Not-Supported*
Resp AI -> Cog Eng(H5a)	0.587	0.033	17.761	0.000	*Supported*
Cog Eng -> INV (H5b)	0.245	0.066	3.708	0.000	*Supported*
Cog Eng -> TNV (H5c)	0.205	0.064	3.413	0.001	*Supported*

*When mediating variable is introduced, Resp AI -> INV and Resp AI -> INV direct effects are suppressed

## Discussion

In this study, we specifically examined the design and implementation of responsible AI in the Indian healthcare context. The mixed-method approach provided insight into the critical constituents of responsible AI in healthcare. The findings revealed three significant pillars of responsible AI in the context of healthcare deliveries: technical skills, ethical concerns, and risk mitigation. Identification of the three facets of responsible AI further divulged exciting aspects of their healthcare implementation. For instance, exploratory interviews revealed that healthcare professionals are becoming competent and skilled in areas other than medicine. This was highlighted as an essential concern as continuous medical education remains an essential priority for most healthcare professionals. The learning requirements associated with responsible AI create a divergent learning track that needs balancing and integration.

Excessive focus on the collection of data was another primary concern emerging from the qualitative study. Respondents indicated that their decisions are increasingly based on recent data, and the department concerned is involved in a continuous evaluation of a large variety of data. The complexity of socially beneficial AI technology necessitates a collaborative effort with technology vendors (Fox & James, 2020). However, the level of trust in these vendors regarding medical data (e.g., fertility, sexual health, fetal diseases, etc.) is significantly low. Some of the interviewees pointed out that while various oaths and medical ethics govern the privacy concerns with respect to healthcare professionals, there is no similar safeguard in respect of technology vendors. The existence of a large number of small and new technology firms as against big and established ones in the domain of responsible AI might also have amplified such privacy concerns.

Further, it emerged from the qualitative study that the algorithmic issues prominent in various healthcare applications are equally important and cannot be ignored. Hence, legislation must safeguard healthcare data and prevent privacy invasion, to ease the leveraging of responsible AI in healthcare. During the qualitative data collection, we also observed that if healthcare professionals are sensitive to risk management activities, their responsiveness towards AI technologies is higher. The exploratory interviews with healthcare professionals helped in understanding the relevance of the constructs under study, as well as identifying its measurement parameters.

The study further utilized a survey of healthcare professionals. The PLS-SEM analysis establishes responsible AI as a third-order formative construct. As expected, we found significant relationships between responsible AI and its underlying dimensions such as technical skills, ethical concerns, and risk mitigation. The specific dimensions of responsible AI bridge the gap between theory and practice by providing insights into socially responsible AI’s design mechanisms. The quantitative data further validated the findings of the qualitative interviews. The empirically validated factors of responsible AI have opened a new digital healthcare arena that can meet the changing laws and regulations on privacy invasion. Such understanding of the components of responsible AI pushes back the existing theoretical knowledge on this domain and provides direction to tackle data security threats. The findings fill the knowledge gaps in this area and offer structured mechanisms to design and implement the responsible AI that meets the stakeholders’ expectations. The study also established these dimensions as second-order reflective constructs. This is the first step to clarify the issues on the formation of responsible AI (Wang et al., 2020). The findings bridge the knowledge gaps in this area and provide a structured mechanism for designing and implementing responsible AI that meets the expectations of the stakeholders. The empirical study could validate the hierarchical model as a reflective–formative model, and the analysis of the structural model confirmed that the paths are significant. In this way, the findings clarified the concepts of reasonable AI, and a valid measurement instrument was developed. As expected and aligned with the exploratory interviews, the quantitative results indicate that risk mitigation is the most crucial factor for developing responsible AI (Ismagilova et al., 2020). One implication of this finding is that despite the skills and competency of healthcare professionals in utilizing health information systems, technology vendors’ design and execution involve a risk. Therefore, it is necessary to consider the risk mitigation activities as an effort in developing responsible AI. Collaborative efforts with NGOs, technical societies, and vendors will ensure a positive social and health impact.

We proposed an integrative framework of responsible AI, cognitive engagement with AI-enabled technologies, instrumental value and terminal value, and market performance. The PLS-SEM analysis results reveal that responsible AI significantly impacts the instrumental value and terminal value (Amit & Zott, 2001). Further, the findings indicate that the intensity of impact on terminal value is more than on instrumental value. Numerous scholars have shown interest in a firm’s value formation activities (Chen et al., 2017; Rokeach, 1973). The study’s findings clarify that responsible AI in healthcare is perceived as satisfactory and good for healthier lives. Besides, responsible AI improves the service quality and delivery mechanisms in healthcare, affecting instrumental value (Zeithmal, 1988). The quantitative assessment also confirmed the partial mediation of cognitive engagement between responsible AI-terminal value and responsible AI-instrumental value. Therefore, patients place significant trust (Fox & James, 2020; Hung et al., 2007)in AI systems that are socially beneficial, provide assurance of data security, and adapt to their fast-changing healthcare needs. The responsiveness of AI-enabled technologies (e.g., assurance of data security, quick recovery of malfunctions and collaborative initiatives for overall health benefits) facilitate patient activation and engagement in self-care and preventive behaviors. Finally, we found that the instrumental value affects market performance, though the relationship between terminal value and market performance was not significant. Our empirical analysis suggests that the instrumental value (increased knowledge about healthcare, level of satisfaction with AI-based service provisions, and the overall healthcare service quality) significantly affects healthcare firms’ market performance (Ravichandran & Lertwongsatien, 2005; Zeithmal, 1988). The findings suggest that development of responsible AI allows healthcare firms to increase market share and introduce new and innovative services.

### Theoretical Implications

Based on our research findings, there are several implications for theory. *First*, our study responds to the recent calls by existing studies (Barello et al., 2016; Reddy, 2018; Shukla & Sushil, 2020; Wang et al., 2020) to identify the constituents of responsible AI. The current study precisely explains how technical skills, ethical concerns, and risk mitigation factors affect responsible AI in the healthcare context. Though much effort has gone into exploring the technological understanding of AI implementation, studies seeking to leverage the ecosystem of responsible AI are scarce. Despite the emerging importance of micro-foundations of psychological underpinnings (Huang & Chang, 2012; Swar et al., 2017), very little is known about the patient’s cognitive engagement with AI and its impact on value formation and market performance. This study fills this knowledge gap.

Second, several versions of the constructs of AI and its antecedents have been utilized in the literature (Duan et al., 2019; Grover et al., 2020; Wang et al., 2018). Many studies across various contexts have outlined different independent variables that could influence responsible AI (Shareef et al., 2021; Wearn et al., 2019). To the contrary, only a few papers are found on what and how responsible AI might influence. Although these noble pursuits invite attention, we examined a wealth of literature relating to responsible AI. This study is the first step to establishing responsible AI as a multi-dimensional third-order construct and investigating a mixed-method approach.

Third, recent researchers have explored the managerial cognitive perspectives of customer benefit and value creation, by considering previous foundations of means-end theory and service-dominant logic (Chen et al., 2017; Skålén et al., 2015). Our empirical studies extend these frameworks by integrating customers’ psychological perspectives and contribute to marketing literature by explaining the dynamics of customer interactions with AI-enabled technologies and service products.

Fourth, the current study’s findings conjoin the IS and marketing literature by investigating the under-represented fields of data privacy, ethical concerns, and co-operative developments and their impacts on market performance. This study also goes beyond the customer-dominant logics of value formation (Brodie et al., 2011; Heinonen et al., 2013), by revealing how responsible AI contributes to their perceived value. Further, our study pushes back the existing frontiers of knowledge concerning technology adoption (Rahman et al., 2016) by the customers and argues that responsible AI systems and artifacts facilitate their cognitive engagement, which is essential from marketing perspectives.

Finally, the findings of our study are also consistent with some previous studies that accept the role of cognitive engagement with different facets of service deliveries and its positive outcomes (Barello et al., 2014; 2016; Graffigana et al., 2015). Our study further explains that cognitive engagement with AI-enabled technologies in healthcare by patients has several benefits, affecting value creation. The study also supports prior studies on data security, privacy invasion, and ethical concerns in healthcare. Of particular note is the study’s finding regarding privacy invasion, quick recovery from malfunctions, and cooperation with technology vendors. The implicit discussions regarding these elements exist in the literature. Only our mixed-method study included these parameters into a comprehensive model and clarified how they are important in implementing responsible AI in healthcare.

### Practical Implications

AI technologies could enable access to a full continuum of care and create an ecosystem with in-home monitoring, acute functionality, and patient assistance for high-value therapies. However, these rapid developments also require technical skills to handle data and algorithms safely, raise individual and service ethics and emphasize risk management. This study highlights the apparent areas of understanding of how responsible AI may be shaped in the healthcare context. The technical challenges relating to data-set appropriateness and suitability and the explainability of algorithms require expertise and team effort. The results suggest that algorithmic issues need much attention to become executable at the frontline of medical care. The study also suggests legitimate data security and privacy invasion avoidance, which are *prima facie* lacking in current Indian healthcare systems. Integration of healthcare provisions with AI technologies throws up an urgent need for healthcare providers and technology vendors to work collaboratively for the best socio-economic outcomes. The study’s findings outline the implementation of responsible AI in healthcare by placing the humans (patients and healthcare professionals) at the center and assess how healthcare companies can be benefitted by the potential healthcare market, as envisaged by the Indian Brand Equity Foundation.

The study’s findings are also aligned with the need for innovations in data science and artificial intelligence, to support global efforts to combat outbreaks of a pandemic like COVID-19 (Al-quaness et al., 2020; Wang et al., 2018). Implementing AI solutions that are socially responsible would enable medical scientists and technologists to address a wide range of biomedical and epidemiological challenges. This study depicts specific steps to tackle COVID-19 challenges by utilizing responsible AI, such as generating public trust through transparency, respecting individual dignity, and facilitating evidence-based clinical decisions (Khalifa et al., 2019; WHO, 2020a). We further argue that understanding cognitive engagement perspectives will provide insights to automate, target, and personalize healthcare marketing activities. For example, the perceived value of wearable devices and other AI-based health monitoring tools will lead to market growth. The fair and safe use of AI tools will increase the patient’s engagement in influencing healthcare firms’ market performance. Our study’s findings shed light on the unexplored ramification of psychological mechanisms of cognitive engagement and provide direction to managers for developing value propositions (e.g., increasing awareness programs, providing assurance of safety, and communicating the convenient use of such AI-based tools).

Further, the study complements and extends the service product perspective by linking the responsible production mechanisms of AI with the patients’ perceived terminal and instrumental value. The rigorous model depicted in this study allows firms to develop appropriate combinations of patient benefits for their AI-enabled tools and platforms. In this way, healthcare firms become patient-centric (Barello et al., 2016), by focusing on marketing capabilities driven by such socially responsible AI technologies.

The study findings suggest that healthcare firms should design service products in general and AI-enabled tools, particularly by taking into account both medical modernization and ethical concerns. The current findings also provide guidelines for healthcare practitioners and policymakers to implement responsible AI for better clinical outcomes, as well as patient benefits such as standard operating procedures for AI tools, regulation for data privacy protection, training to improve technical skills, reducing harmfulness, quick actions for recovery, and conducting audits of the transparency. Our study results suggest that healthcare policymakers should adopt a broader perspective of strategic choices and abandon the rigid delivery models. Moreover, the findings suggest how healthcare managers can focus on developing dynamic cognitive capabilities (Almquist et al., 2016) to identify responsible AI’s perceived benefit and its linkage with value formation. Further, the model proposed in this study may also facilitate scrutiny of healthcare practices and offer value propositions through a wide array of AI-enabled technologies that are socially responsible. From the patients’ perspective, cognitive engagement with AI-enabled technologies will facilitate positive perceptions and create a level of trust while interacting with them.

### Social Implications

The digital divide concerns and its possible negative social implication on society’s vulnerable sections have always remained a critical apprehension for researchers. Efforts to solve this challenge in the context of an emerging technology must be made since the initial days of its conceptualization. Responsible AI is, in fact, an attempt to solve this social challenge of equitable distribution of benefits. By identifying dimensions of responsible AI, this study would help the citizens group in two key ways. First, by identifying the list of possible vulnerabilities like discrimination, autonomy, and privacy invasion, this study would help them understand the possibility of unequal distribution of benefits from AI application in the domain of healthcare. Second, by identifying risk mitigating strategies like adaptability, cooperation, and transparency, this study would further help the citizens group in directing their resources towards established and scientifically proven managerial solutions. Active citizen groups working towards the cause of equitable access to healthcare facilities would further benefit by learning various aspects of applying responsible AI in healthcare.

Similarly, the managers and owners of healthcare firms would benefit from a better understanding of responsible AI. They would be able to serve the diverse population with effective and efficient healthcare offerings. This study would also equip them with knowledge of balancing growth and sustainability, thus providing long-term benefits to society. The study would further guide the younger generation in identifying the requirement of skills in these emerging domains – the scope for which is expected to follow a sharp upward trajectory. It will thus help them in preparing themselves for the future job market. The findings suggest how the responsible AI solutions can lower the barriers between hospitals and patients and improve access to care, particularly in tier II and tier III cities of India. The findings also provide ways to develop a vibrant start-up ecosystem that goes beyond the clinical services to a new business model of wellness, prevention, monitoring services, even in non-metro cities of India.

### Limitations and Future Research Avenues

The present study, though it has certain limitations, generates some avenues for future research. First, the study was conducted in an Indian context. Future research should test the framework’s generalizability in contrasting contexts (emerging country healthcare) to provide a robust understanding. A fruitful avenue of future research is the extension of the framework by considering the cultural characteristics and collecting the multi-national samples to assess the strength of the relationships. Second, the study contributes to the constituents of ‘Responsible AI’ in healthcare by considering several psychological and social aspects. However, there may be other contextual variables affecting the formation of Responsible AI, which remain unexplored. Future studies should also be conducted to better understand the antecedents of responsible AI. Third, we believe that the nature of tasks and the degree of involvement with AI-enabled technology may also moderate this relationship. For example, different users may use different tools and platforms as per their requirements. Task-related and rational motives may affect the perceived benefits and value differently. Future researchers should take into account the moderating roles of such variables and explore these issues of personal habits and task characteristics, which the current study has not considered. Finally, theories of psychology suggest that the factors responsible for cognitive engagement are affected by the brands of service products, and may also change over time. Therefore, a longitudinal study considering the brand values of the AI-enabled technologies will provide more useful insights to strengthen the theorizing in this context.

## Conclusions

The widespread interest in responsible AI shown by scholars and practitioners motivated this research article. Although several industry reports and contributions of practitioners have populated the literature on responsible AI, researchers have only recently begun exploring its underlying dynamics. In essence, this study provides a background to the infrastructure and ecosystem that supports the formation of responsible AI in the healthcare context. This study highlights the dark side that a typical AI project could present and thus addresses an urgent need to develop AI technologies with social and ethical concerns. In doing so, we adopted a mixed methodology, using a sample of both healthcare professionals and customers (patients) in India. Our results first identify the constituents of responsible AI and establish it as a third-order factor with three underlying dimensions: technical skills, ethical concerns, and risk-mitigation. We found that data and algorithmic issues, privacy invasion, adaptability, quick recovery from malfunctions, and collaborative efforts were important in shaping responsible AI in healthcare. The insights of the study can be used for easing the leveraging of responsible AI in India. Our findings guide healthcare firms in designing and implementing responsible AI, while simultaneously clarifying how such technological advancements affect patients’ cognitive engagement. Our findings provide the insight that responsible AI affects the value formation and market performance of a healthcare firm.

## Appendix

**Table 9:** Interview agenda

Sl. No.	Interview agenda
1	Prevalence of AI based applications in hospital
2	History of implementation of AI based solutions
3	Process of skill development for healthcare professionals
4	Various challenges of implementation
5	Ethical concerns (possibility and past complaints)
6	Types of ethics and ways to handle them
7	Trust between various stakeholders
8	Privacy concerns
9	Evidence-based medicine and AI
10	Reducing malfunctions
11	Role of the technology partner and relationships with them
12	Risk associated with AI and how does it differ from other technologies
13	Cost and benefits from AI

**Table 10:** Respondent’s profile in qualitative study

Sl. No.	Role and profile	Number of interviews	Approximate duration
1	Medical Director, 61, Male	4	160 min
2	Medical IT Officer, 49, Male	4	180 min
3	Medical Dean, 58, Female	4	180 min
4	Chief Operating Officer, 52, Male	4	160 min
5	Senior Consultant, 47, Male	1	60 min
6	Senior Consultant, 43, Female	1	50 min
7	Senior Consultant, 59, Male	1	50 min
8	Senior Consultant, 51, Female	1	50 min
9	Consultant, 40, Male	1	60 min
10	Consultant, 41, Female	1	60 min
11	Chief Administrative officer, 44, Male	2	100 min
12	Medical Record Officer, 37, Male	2	120 min

**Table 11:** Measurement scale

Measurement items	Reference
Data	(Gupta & George, 2016)
We integrate data for high-value analysis for our business environment (DD1)	
We continuously assess our data for responsible execution (DD2)**	
Our decisions are based on data rather than our instinct (DD3)	
We include fairness in data selection (DD4)	
We use the data set that are relevant to the population (DD5)	
Algorithm	(Al-quaness et al., 2020)
We ensure that the design of our algorithms should be explainable (Alg1)**	
We continuously attempt to reduce algorithm biases (Alg2)	
We have a monitoring system for the development of AI systems (Alg3)	
We are sensitive to make the algorithms robust (Alg4)	
Individual Ethics	
We are impartial and avoid unfair discrimination (INE1)	
We avoid violation of trust (INE2)	
We deal fairly with people (INE3)	
We respect each person’s autonomy (INE4)	
Service-ethics	
We maintain privacy invasion (SRE1) **	
We conceive the AI only after ensuring sufficient understanding of purposes (SRE2)	
We ensure that the responsible entity is apparent (SRE3)	
We conduct an impact assessment to ensure less harmful ways of achieving the objective (SRE4)	
Adaptability	(Fox & James, 2020)
We ensure the adaptive performance of intended functions (ADT1)	
We quickly reconfigure our programs as per the requirements(ADT2)	
We resist processing when unexpected harm arises (ADT3)	
We recover when malfunctions occur (ADT4)	
Co-operation	(Fox & James, 2020)
We advocate a more thorough mapping of possible collaboration (Cop1)	
We associate with our partners to work in a collaborative environment (Cop2)**	
We take collective action on responsible AI development (Cop3)	
Transparency	(Fox & James, 2020)
AI-based processes are transparent to all stakeholders (TRP1)	
We ensure that the people are aware of actions and inferences (TRP2)	
All entities have obligations to procedural fairness (TRP3)	
Market Performance (MP)	(Ravichandran & Lertwongsatien, 2005)
We have entered a new market quickly (MKP1)	
We have added news services for the patients quickly (MKP2)	
Our success rate is higher as compared to our competitors (MKP3)	
Our market share has increased (MKP4)	
Cognitive Engagement (Cog Eng)	(Graffigana et al., 2015)
I feel comfortable in utilizing AI technologies (Cog1)	
I understand that AI-enabled platforms are essential for health- development (Cog2)	
I believe that AI enabled tools are not risky (Cog3)	
The features offered by AI-enabled tools can be adjusted to fit our need (Cog4)	
Instrumental value (INV)	(Chen et al., 2017)
The use of the AI-enabled platform can increase our abilities (INV1)	
The use of the AI-enabled platform can increase our imagination (INV2)	
The use of the AI-enabled platform can inspire our curiosity (INV3)	
The use of the AI-enabled platform can increase our knowledge (INV4)	
Terminal value (TNV)	(Chen et al., 2017)
The use of the online streaming platform helps you feel relaxed and happy (TNV1)	
The use of the online streaming platform enhances your confidence (TNV2)	
Family feelings become better after using the online streaming platform (YNV3)	
The use of the online streaming platform matures your view of life (TNV4)	

**Exploratory interviews output

## References

[CR1] Abosaq NH (2019). Impact of privacy issues on smart city services in a model smart city. International Journal of Advanced Computer Science and Applications.

[CR2] Agarwal R, Anderson C, Zarate J, Ward C (2013). If we offer it, will they accept? Factors affecting patient use intention of personal health records and secure messaging. Journal of Medical Internet Research.

[CR3] AHHM. (2017). *India Digital Health Report 2017*. Retrieved from https://www.asianhhm.com/healthcare-reports/india-digital-health-report.

[CR4] Ahmed, M. A., Eckert, C., & Teredesai, A. (2018). Interpretable machine learning in healthcare. *International Conference on Bioinformatics, Computaional Biology, and Health Informatics*, pp. 559–560.

[CR5] Almquist E, Senior J, Bloch N (2016). The elements of value. Harvard Business Review.

[CR6] Al-quaness M, Ewees AA, Fan AA, Aziz AE (2020). Optimization method for forecasting confirmed cases of COVID-19 in China. Journal of Clinical Medicine.

[CR7] Amit R, Zott C (2001). Value creation in E-business. Stategic Management Journal.

[CR8] Amoore L, Raley R (2017). Securing with algorithms: Knowledge, decision, sovereignty. Security Dialogue.

[CR9] Bag S, Pretorius JHC, Gupta S, Dwivedi YK (2021). Role of institutional pressures and resources in the adoption of big data analytics powered artificial intelligence, sustainable manufacturing practices and circular economy capabilities. Technological Forecasting and Social Change.

[CR10] Balakrishnan, J., & Dwivedi, Y. K. (2021). Role of cognitive absorption in building user trust and experience. *Psychology & Marketing*, 1–26, 10.1002/mar.21462.

[CR11] Barello S, Graffigna G, Vegni E, Bosio AC (2014). The challenges of conceptualizing patient engagement in health care: A lexicographic literature review. The Journal of Participatory Medicine.

[CR12] Barello S, Triberti S, Graffigna G, Libreri C, Serino S, Hibbard J, Riva G (2016). eHealth for patient engagement: A systematic review. Frontiers in Psychology.

[CR13] Baron R, Kenny D (1986). The moderator-mediator variable distinction in social psychological research: Conceptual, strategic, and statistical considerations. Journal of Personality and Social Psychology.

[CR14] Barrat, J. (2013). *Our final invention: Artificial Intelligence and the end of the human era*. Thomas Dunne Books.

[CR15] Bashshur, R., Shannon, G., Krupinski, E., & Grigsby, J. (2011). The taxonomy of telemedicine. *Telemedicine and E-Health*, *17*(484–494). 10.1089/tmj.2011.0103. 10.1089/tmj.2011.0103 21718114

[CR16] Basu, A., Mehta, R., & Majumdar, A. (2021). State of healthcare in India. Retrieved February 25, 2021, from PwC India website: https://www.pwc.in/industries/healthcare/reimagining-the-possible-in-the-indian-healthcare-ecosystem-with-emerging-technologies.html.

[CR17] Bate, P., & Robert, G. (2007). *Bringing user expereince to Healthcare Improvement*. Radcliffe Publishing Limited.

[CR18] Bengatson M, Kock S (2000). “coopetition” in business networks- to coperate and compete simulataneously. Industrial Marketing Management.

[CR19] Bichinadaritz I, Marling C (2006). Case-based reasoning in the health sciences: What’s next?. Artificial Intelligence in Medicine.

[CR20] Brisimi T, Chen R, Mela T, Ch. Paschalidis AO, Shi W (2018). Federated learning of predictive models from federated Electronic Health Records. International Journal of Medical Informatics.

[CR21] Brodie RJ, Hollebeek LD, Juric B, Ilic A (2011). Customer engagement: Conceptual domain, fundamental propositions, and implications for research. Journal of Service Research.

[CR22] Brozovic D, Nordin F, Kindstrom D (2016). Service flexibility: conceptualizing value creation in service. Journal of Service Theory and Practice.

[CR23] Burkhardt, R., Hohn, N., & Wigley, C. (2019). *Leading your organization to responsible AI*.

[CR24] Campbell JL (2007). Why would corporations behave in socially responisble way? an institutional theory of corporate social responsibility. Academy of Management Review.

[CR25] Chace, C. (2015). *Surviving AI: The promise and Peril of Artificial Intelligence* (Bradford, Ed.). Three Cs.

[CR26] Chatterjee S (2020). AI strategy of India: policy framework, adoption challenges and actions for government. Transforming Government: People, Process and Policy.

[CR27] Chen, N. (2018). *Are robots replacing routine jobs ?* Cambridge, M.A.

[CR28] Chen Y, Liu H, Chiu Y (2017). Customer benefits and value creation in streaming services marketing: a managerial cognitive capability approach. Psychology & Marketing.

[CR29] Chopra K (2019). Indian Shoppers motivation to use artificial intelligence: generating vroom’s expectancy theory of motivation using grounded theory approach. International Journal of Retail and Distribution Management.

[CR30] Chronbach LJ (1951). Coefficient alpha and the internal structure of tests. Psychometrika.

[CR31] Coulter A, Ellins J (2007). Effectiveness of strategies for informing, educating, and invoving patients. British Medical Journal.

[CR32] Creswell, J. W. (2006). *Qualitative enquiry and research design: Choosing among five approaches* (2nd ed.). Sage.

[CR33] Da Silva AS, Farina MC, Gouvea MA, Denis D (2015). A Model of antecedents for the co-creation of value in healthcare: An application of structure equation modeling. Brazilian Bussiness Review.

[CR34] Daugherty PR, Wilson HJ, Chowdhury R (2019). Using artificial intelligence to promote diversity. MT Sloan Management Review.

[CR35] De Sarbo WS, Jedidi K, Sinha I (2001). Cutomer value analysis in a heterogenous market. Strategic Management Journal.

[CR36] Deven RD, Joshua AK (2017). A guide to algorithms and the law. Harvard Journal of Law & Tchnology.

[CR37] Doumbouya M, Kamsu-foguem B, Kenfack H, Foguem C (2014). Telematics and Informatics Telemedicine using mobile telecommunication: Towards syntactic interoperability in teleexpertise. Telematics and Informatics.

[CR38] Duan Y, Edwards JS, Dwivedi YK (2019). Artificial intlligence for decision making in the era of big data- evolution, challenges and research agenda. International Journal of Information Management.

[CR39] Dubey, R., Bryde, D. J., Foropon, C., Tiwari, M., Dwivedi, Y., & Schiffling, S. (2020). An investigation of information alignment and collaboration as complements to supply chain agility in humanitarian supply chain. *International Journal of Production Research*, 1–20. 10.1080/00207543.2020.1865583.

[CR40] Dwivedi YK, Hughes L, Ismagilova E, Aarts G, Coombs C, Crick T, Williams MD (2021). Artificial Intelligence (AI): Multidisciplinary perspectives on emerging challenges, opportunities, and agenda for research, practice and policy. International Journal of Information Management.

[CR41] Dwivedi, Y. K., Ismagilova, E., Hughes, D. L., Carlson, J., Filieri, R., Jacobson, J., … Wang, Y. (2020). Setting the future of digital and social media marketing research: Perspectives and research propositions. *International Journal of Information Management, 102168*. 10.1016/j.ijinfomgt.2020.102168.

[CR42] Eggers JP, Kaplan S (2013). Cognition and capabilities: A multi-level perspective. Academy of Management Annals.

[CR43] Faber S, Geenhuizen M, Van, De Reuver M (2017). eHealth adoption factors in medical hospitals: A focus on the Netherlands. International Journal of Medical Informatics.

[CR44] FHI. (2020). *The age of opportunity: Empowering the next generation to transform healthcare*. Retrieved from https://www.philips.co.in.

[CR45] Fornell C, Larcker DF (1981). Evaluating structural equation models with unobservable variables and measurement error. Journal of Marketing Research.

[CR46] Fox G, James TL (2020). Toward an understanding of the antecedents to health information privacy concern: A mixed methods study. Information Systems Frontiers.

[CR47] Fuentes C (2015). Images of responsible consumers: organizing the marketing of sustainability. International Journal of Retail & Distribution Management.

[CR48] Gambhir S, Malik S, Kumar Y (2016). Role of soft computing approaches in healthcare domain: A mini review. Journal of Medical Systems.

[CR49] GDPR. (2019). Data protection rules as a trust -enabler in the EU and beyond- taking stock.

[CR50] Gen L (2015). Forecast enrollment rate in clinical trials. Applied Clinical Trials.

[CR51] Ghallab M (2019). Responsible AI: requirements and challenges. AI Perspectives.

[CR52] Gooty J, Francis JY (2011). Dyads in organizational research: Conceptual issues and multilevel analyses. Organizational Research Methods.

[CR53] Graffigana G, Barello S, Bonanomi A, Lozza E (2015). Measuring patient engagement: developement and psychometric properties of the Patient Health Engagement (PHE) Scale. Frontiers in Psychology.

[CR54] Gronroos C, Gummerus J (2014). The service revolution and its marketing implications: service logic vs service-dominant logic. Managing Service Quality.

[CR55] Gronroos C, Ravald A (2010). Service as business logic: implications for value creation and marketing. Journal of Service Management.

[CR56] Grover, P., Kar, A. K., & Dwivedi, Y. K. (2020). Understanding artificial intelligence adoption in operations management: insights from the review of academic literature and social media discussions. In *Annals of Operations Research*. 10.1007/s10479-020-03683-9.

[CR57] Gummenson E (2005). Qualitative research in marketing: road-map for a wilder-ness of complexity and unpredictability. Europian Journal of Marketing.

[CR58] Gupta M, George JF (2016). Toward the development of a big data analytics capability. Information & Management.

[CR59] Gursoy D, Chi OH, Lu L, Nunkoo R (2019). Consumers acceptance of artificially intelligent (AI) device use in service delivery. International Journal of Information Management.

[CR60] Hair, J. F., Hult, J., Ringle, G. T. M., & Sarstedt, M. (2016). *A primer on partial least least square structural equation modeling (PLS-SEM)* (2nd ed.). Sage.

[CR61] Harrison RL, Reilly TM (2011). Mixed methods designs in marketing research. Qualitative Market Research: An International Journal.

[CR62] He J, Baxter SL, Xu J, Xu J, Zhou X, Zhang K (2019). The practical implementaion of artificial intelligence technologies in medicine. Nature Medicine.

[CR63] Heinonen K, Strandvik T, Voima P (2013). Customer dominant value formation in service. European Business Review.

[CR64] Henseler, J., Dijkstra, T. K., Sarsteadt, M., Ringle, C. M., Diamantopoulos, A., Straub, D. W., & Calantone, R. J. (2014). Common beliefs and reality about PLS: Comments on Ronkko and Evermann (2013). *Organizational Research Methods*, *17*(2), 182–209.

[CR65] Hibbard JH, Mahoney ER, Stock R, Tusler M (2007). Do increases in patient activation result in improved self-management behaviour?. Health Services Research.

[CR66] Hota C, Upadhyaya S, Al-karaki JN (2015). Advances in secure knowledge management in the big data era. Information Systems Frontier.

[CR67] Hsu, W. C. J., Liou, J. J. H., & Lo, H. W. (2021). A group decision-making approach for exploring trends in the development of the healthcare industry in Taiwan. *Decision Support Systems*, *141*(May 2020), 113447. 10.1016/j.dss.2020.113447.

[CR68] Huang E, Chang CC (2012). Patient-oriented ineractive e-health tools on U.S. hospital web sites. Health Marketing Quarterly.

[CR69] Hung PCK, Chiu DKW, Fung WW, Cheung WK, Wong R, Choi SPM, Cheng VSY (2007). End-to-end privacy control in service outsourcing of human intensive processes: A multi-layered Web service integration approach. Information Systems Frontiers.

[CR70] Ismagilova, E., Hughes, L., Rana, N. P., & Dwivedi, Y. K. (2020). Security, privacy and risks within smart cities: Literature review and development of a smart city interaction framework. *Information Systems Frontiers*. 10.1007/s10796-020-10044-1. 10.1007/s10796-020-10044-1 PMC7373213 32837262

[CR71] Javalgi RG, Whipple, Thomas W, Ghosh AK, Young RB (2005). Market orientation, strategic flexibility, and performance : implications for services providers. Journal of Services Marketing.

[CR72] Johnson JL, Lee RP, Grohmann B (2003). Market-focused strategic flexibility: Conceptual advances and an integrative model. Journal of Academy of Marketing Science.

[CR73] Joubert, A., Murawski, M., & Bick, M. (2021). Measuring the big data readiness of developing countries – index development and its application to Africa. *Information Systems Frontiers*, (2020). 10.1007/s10796-021-10109-9.

[CR74] Khalifa M, Magrabi F, Gallego B (2019). Developing aframework for evidence-based grading and assessment of predictive tools for clinical decision support. BMC Medical Informatics and Decision Making.

[CR75] Khanna S, Sattar A, Hansen D (2012). Advances in artificial intelligence research in health. Australasian Medical Journal.

[CR76] Kim E-Y (2015). Patient will see you now: The future of medicine is in your hands. Healthcare Informatics Research.

[CR77] Kok, J., Kosters, E. J. W. B., Van Der, W., P. P., & Poel, M. (2013). Artificial intelligence: definition, treds, techniques, and cases. In *Encyclopedia of Life Support Systems*. UK: Oxford,UK.

[CR78] Leslie S (2019). Data-from objects to assets. Nature.

[CR79] Linn AJ, Vervloet M, Dijk V, Smit EG, Van Weert JC (2011). Effects of eHealth interventions on medication adherence: a systematic review of the literature. Journal of Medical Internet Research.

[CR80] Lui A, Lamba GW (2018). Artificial intelligence and augmented intelligence collaboration: regaining trust and confidence in the financial sector. Journal of Information and Communications Technology Law.

[CR81] Lusch R, Nambisan S (2015). Service innovation: a service- dominant logic perspective. MIS Quarterly.

[CR82] Magids S, Zorfas A, Leemon D (2015). The new science of customer emotions. Harvard Business Review.

[CR83] Malhotra NK, Kim SS, Patil A (2006). Common method variance in IS research: A comparison of alternative approaches and a reanalysis of past research. Management Science.

[CR84] Manyika, J., Chui, M., Bughin, J., Dobbs, R., Bisson, P., & Marrs. (2013). Disruptive technologies: Advances that will transform life, business, and the global economy. In *McKinsey Global Insitute*.

[CR85] Marano A, Di Nicolantonio M (2015). Ergonomic design in eHealthcare: a study case of ehealth technology system. Procedia Manufacturing.

[CR86] Markets, I. (2020). *Digital Healthcare in India “Healthcare of the Future.”* Retrieved from https://www.indiahealth-exhibition.com/content/dam/Informa/indiahealth-exhibition/en/downloads/Digitalhealthreport2020.pdf.

[CR87] MCI. (2016). *Privacy Policy in Healthcare: Policy Guide*. New Delhi.

[CR88] Mikalef P, Gupta M (2021). Artificial intelligence capability: Conceptualization, measurement calibration, and empirical study on its impact on organizational creativity and firm performance. Information & Management.

[CR89] Modjarrad K, Moorthy VS, Miller P, Gsell PS, Roth C, Kieny MP (2016). Developing global norms for sharing data and results during public health emergencies. Plos Medicine.

[CR90] NAH. (2020). Future of AI in healthcare in India.

[CR91] Nair A, Nicolae M, Narasimhan R (2013). Examining the impact of clinical quality and clinical flexibility on cardiology unit performance — Does experiential quality act as a specialized complementary asset ?. Journal of Operations Management.

[CR92] NeHA. (2016). *Concept Note- National e-Health Authority (NeHA)*. New Delhi.

[CR93] Nishant R, Kennedy M, Corbett J (2020). Artificial intelligence for sustainability: Challenges, opportunities, and a research agenda. International Journal of Information Management.

[CR94] NITI Aayog. (2016). NITI Aayog leads initiative to convert 100 % Government – Citizen Transactions to the digital platform. Retrieved August 8, 2017, from http://niti.gov.in/content/digital-payments.

[CR95] Obermeyer Z, Powers B, Vogeli C, Mullainthan S (2019). Dissecting racial bias in an algorithm used to manage the health of population. Science.

[CR96] OECD. (2019). Artificial intelligence in society. 10.1787/eedfee77-en.

[CR97] Parry, M. E. (2001). *Strategic marketing management*. McGraw-Hill.

[CR98] Pillai R, Sivathanu B, Dwivedi YK (2020). Shopping intention at AI-powered automated retail stores (AIPARS). Journal of Retailing and Consumer Services.

[CR99] Pillai, R., Sivathanu, B., Mariani, M., Rana, N. P., Yang, B., & Dwivedi, Y. K. (2021). Adoption of AI-empowered industrial robots in auto component manufacturing companies. Production Planning & Control, 1–17, 10.1080/09537287.2021.1882689.

[CR100] Porra J, Lacity M, Parks MS (2020). Can computer based human-likeness endanger humanness? – A philosophical and ethical perspective on digital assistants expressing feelings they can’t have. Information Systems Frontiers.

[CR101] Rahman MS, Ko M, Warren J, Carpenter D (2016). Healthcare Technology Self-Efficacy (HTSE) and its influence on individual attitude: An empirical study. Computers in Human Behavior.

[CR102] Rana NP, Dwivedi YK (2016). Using clickers in a large business class: Examining use behavior and satisfaction. Journal of Marketing Education.

[CR103] Ravichandran T, Lertwongsatien (2005). Effect of information systems resources and capabilities on firm perofrmance: a resource-based perspective. Journal of Management and Information Systems.

[CR104] Reddy, S. (2018). Use of artifical intelligence in healthcare Delivery. In *eHealth- Making Health Care Smarter* (pp. 81–97). IntechOpen.

[CR105] Ringle, C. M., Wende, S., & Becker, J. (2017). *SmartPLS 3*.

[CR106] Rokeach, M. (1973). *The nature of human values*. The Free Press.

[CR107] Saha E, Ray PK (2019). Modelling and analysis of inventory management systems in healthcare: A review and reflections. Computers & Industrial Engineering.

[CR108] Sergio, A. (2015). *A model of antecedents for the co-creation of value in health care: an application of structural equation modeling*. (11), 121–149.

[CR109] Serino, S., Triberti, S., Villani, D., Cipresso, P., & Doherty, G. (2014). Toward a validation of cyber-interventions for stress disorders based on stress inoculation training: a systematic review. *Virtual Real*, *18*. 10.1007/s10055-013-0237-6.

[CR110] Shaikhina T, Khovanova NA (2017). Handling limited datasets with neural networks in medical applications. Artificial Intelligence in Medicine.

[CR111] Shareef, M. A., Kumar, V., Dwivedi, Y. K., Kumar, U., Akram, M. S., & Raman, R. (2021). A new health care system enabled by machine intelligence: Elderly people’s trust or losing self control. *Technological Forecasting and Social Change*, *162*(August 2020), 120334. 10.1016/j.techfore.2020.120334.

[CR112] Sharma SK (2019). Integrating cognitive antecedents into TAM to explain mobile banking behavioral intention: A SEM-neural network modeling. Information Systems Frontiers.

[CR113] Sharma SK, Al-Badi AH, Govindaluri SM, Al-Kharusi MH (2016). Predicting motivators of cloud computing adoption: A developing country perspective. Computers in Human Behavior.

[CR114] Sharma SK, Gaur A, Saddikuti V, Rastogi A (2017). Structural equation model (SEM)-neural network (NN) model for predicting quality determinants of e-learning management systems. Behaviour and Information Technology.

[CR115] Sharma SK, Sharma M (2019). Examining the role of trust and quality dimensions in the actual usage of mobile banking services: An empirical investigation. International Journal of Information Management.

[CR116] Shukla, S. K., & Sushil (2020). Evaluating the practices of flexibility maturity for the software product and service organizations. *International Journal of Information Management*, *50*(April 2019), 71–89. 10.1016/j.ijinfomgt.2019.05.005.

[CR117] Singhal, S., & Carlton, S. (2019). *The era of exponential improvement in healthcare?*

[CR118] Sivarajah U, Kamal M, Irani Z, Weerakkody V (2017). Critical analysis of big data challenges and analytical methods. Journal of Business Research.

[CR119] Skålén P, Gummerus J, Koskull C, Von, Magnusson PR (2015). Exploring value propositions and service innovation: a service-dominant logic study. Journal of the Academy of Marketing Science.

[CR120] Sultan N (2015). Reflective thoughts on the potential and challenges of wearable technology for healthcare provision and medical education. International Journal of Information Management.

[CR121] Swar B, Hameed T, Reychav I (2017). Information overload, psychological ill-being, and behavioral intention to continue online healthcare information search. Computers in Human Behavior.

[CR122] Tenehaus, M., Vinzi, V. E., Chatelin, Y. M., & Lauro, C. (2005). Computational statistics & data analysis. In *PLS Path Modeling* (pp. 159–205).

[CR123] Thomas, S. (2020). *State of Artificial Intelligence in India*. New Delhi.

[CR124] Tyagi, H. (2019). Digital health start-ups in India: The challenge of scale. Retrieved February 1, 2021, from Forbes India website: https://www.forbesindia.com/article/isbinsight/digital-health-startups-in-india-the-challenge-of-scale/52799/1.

[CR125] Vayena, E., Blasimme, A., & Cohen, I. G. (2018). Machine learning in medicine: Addressing ethical challenges. *Plos Medicine, 15*(11), e1002689. 10.1371/journal.pmed.1002689 PMC6219763 30399149

[CR126] Vellido A (2019). The importance of interpretability and visualization in machine learning for applications in medicine and health care. Neural Computing and Applications.

[CR127] Venkatesh V, Brown SA, Bala H (2013). Bridging the qualitative and quantitative divide: Guidelines for conducting mixed methods research in information systems. MIS Quarterly.

[CR128] Vinzi, V. E., Trinchera, L., & Amato, S. (2010). PLS path modeling: from foundations to recent developments and open issues for model assessment and improvement. In *Handbook of Partial Least Squares* (pp. 47–83). Springer.

[CR129] Wang N, Liang H, Zhong W, Xue Y, Xiao J (2012). Resource structuring of or capability buiding? An empirical study of the business value of information technology. Journal of Management Information System.

[CR130] Wang Y, Kung L, Byrd TA (2018). Big Data Analytics: Understanding its capabilities and potential benefits for healthcare organizations. Technological Forecasting & Social Change.

[CR131] Wang, Y., Xiong, M., & Olya, H. (2020). Toward an understanding of responsible artificial intelligence practices. *53rd Hawaii Internationational Conference on System Sciences*. Maui, Hawaii, USA.

[CR132] Warwick, K. (2013). *Artificial Intelligence: The Basics*. Routledge.

[CR133] Wearn OR, Freeman R, Jacoby DM (2019). Responsible AI for conservation. Nature Machine Intelligence.

[CR134] WHO. (2020a). *COVID-19 updates from India*. New Delhi.

[CR135] WHO. (2020b). Guidelines on digital health interventions. Retrieved January 27, 2021, from World Health Organization website: https://www.who.int/news/item/17-04-2019-who-releases-first-guideline-on-digital-health-interventions.

[CR136] Wimmer H, Yoon V, Sugumaran V (2016). A multi-agent system to support evidence based medicine an clinical decision making via data sharing and data privacy. Decision Support Systems.

[CR137] Winter JS, Davidson E (2019). Governanace of artificial intelligence and personal health information. Digital Policy, Regulation and Governance.

[CR138] Wu, H., Deng, Z., Wang, B., & Wang, H. (2021). How online health community participation affects physicians’ performance in hospitals: Empirical evidence from China. *Information & Management*, *103443*. 10.1016/j.im.2021.103443.

[CR139] Zeithmal VA (1988). Consumer perception of price, quiality, and value: A means-end model and synthesis of evidence. Journal of Marketing.

[CR140] Zink, A., & Rose, S. (2020). Fair Regression for Health Care Spending. *Biometrics*, *biom.13206*. 10.1111/biom.132026. 10.1111/biom.13206 PMC7540596 31860120

[CR141] Zuboff S (2015). Big other: Surveillance capitalism and the impact of an information civilization. Journal of Information Technology.

